# Crystal structure of the modification-dependent SRA-HNH endonuclease TagI

**DOI:** 10.1093/nar/gky781

**Published:** 2018-09-07

**Authors:** Marlena Kisiala, Alyssa Copelas, Honorata Czapinska, Shuang-yong Xu, Matthias Bochtler

**Affiliations:** 1 International Institute of Molecular and Cell Biology, Trojdena 4, 02-109 Warsaw, Poland; 2 Institute of Biochemistry and Biophysics PAS, Pawinskiego 5a, 02-106 Warsaw, Poland; 3 Biological and Chemical Research Centre, University of Warsaw, Zwirki i Wigury 101, 02-089 Warsaw, Poland; 4 New England Biolabs, Inc. 240 County Road, Ipswich, MA 01938, USA

## Abstract

TagI belongs to the recently characterized SRA-HNH family of modification-dependent restriction endonucleases (REases) that also includes ScoA3IV (Sco5333) and TbiR51I (Tbis1). Here, we present a crystal structure of dimeric TagI, which exhibits a DNA binding site formed jointly by the nuclease domains, and separate binding sites for modified DNA bases in the two protomers. The nuclease domains have characteristic features of HNH/ββα-Me REases, and catalyze nicks or double strand breaks, with preference for /RY and RYN/RY sites, respectively. The SRA domains have the canonical fold. Their pockets for the flipped bases are spacious enough to accommodate 5-methylcytosine (^5m^C) or 5-hydroxymethylcytosine (^5hm^C), but not glucosyl-5-hydroxymethylcytosine (^g5hm^C). Such preference is in agreement with the biochemical determination of the TagI modification dependence and the results of phage restriction assays. The ability of TagI to digest plasmids methylated by Dcm (C^5m^CWGG), M.Fnu4HI (G^5m^CNGC) or M.HpyCH4IV (A^5m^CGT) suggests that the SRA domains of the enzyme are tolerant to different sequence contexts of the modified base.

## INTRODUCTION

Modification of DNA is commonly occurring in phages, and examples have been found for variants of all four standard DNA bases in their genomes. Modified bases include 5-methylcytosine (^5m^C), N4-methylcytosine (^4m^C), N6-methyladenine (^6m^A), 5-hydroxymethyluracil (^5hm^U) and 5-hydroxymethylcytosine (^5hm^C), their glucosylated variants (^g5hm^U and ^g5hm^C), as well as several other already demonstrated or predicted modified bases (1–4). Mechanistically, phages or other mobile elements can acquire DNA modifications by passage in the host, or by incorporation of modified dNTPs into DNA, which can then be optionally modified further. Cytosine C5 and N4 methylation, as well as adenine N6 methylation can be acquired after DNA synthesis in the host, or as a result of the activity of orphan phage-encoded DNA methyltransferases (MTases) (5). Other nucleobases like ^5hm^U and ^5hm^C are incorporated directly by polymerase processing modified triphosphates (6). Modified bases in phage or other mobile DNA provide protection against ‘conventional’ host restriction. For example, 2′-deoxyguanosine replacement by 2′-deoxyarchaeosine (dG^+^) in the *Escherichia coli* phage 9g DNA renders it resistant to over 70% of commercially available Type II restriction endonucleases (REases) (7). Similarly, α-putrescinylthymine (putT) in phi W-14 DNA has been found to block DNA cleavage by more than half of all tested Type II REases (8). In addition to protection against conventional restriction systems, modified DNA bases may also have other functions, for example may facilitate packing of DNA in the phage head (9).

In response to the emergence of phages with modified DNA, some bacteria have evolved REases that are directed against such substrates. So far, examples have been found for enzymes that specifically target modified adenine and cytosine bases. Even for these bases, the modifications that are shown to direct REase cleavage constitute only a subset of the full repertoire of known modifications. Until now, only ^6m^A, ^5m^C, ^5hm^C and ^g5hm^C have been shown to be targetable. Promiscuity for both adenine and cytosine methylation has been inferred for Mrr from *E. coli* based on genetic data, but not yet demonstrated *in vitro* (10). Otherwise, modification-dependent REases appear to be specific for either modified adenine or cytosine, but not both. Methyladenine-dependent restriction has been clearly demonstrated only for DpnI, a Type IIM enzyme with specificity for G^6m^ATC target sequence (11,12). In addition, ^6m^A is known to play a role in phage growth limitation (Pgl) systems, but the hypothesized ^6m^A-dependent REase has not yet been identified (13). Some cytosine modification dependent REases cleave highly GC-rich target sequences containing several modified cytosines directly within the recognition sequence (GlaI (14), BisI (15), Eco15I (15) and EcoBLMcrX (16)). However, more typically, cytosine modification dependent REases cleave at considerable distance from one or more modified cytosine bases. These enzymes can be further divided into a group of nucleotide triphosphate dependent, hetero-oligomeric enzymes (McrBC (17–19), GmrSD (20,21), SauUSI (22)) and a group of nucleotide triphosphate independent homo-oligomeric enzymes.

The methylcytosine dependent, remote site cleaving, NTP-independent REases include the McrA (RglA) (23,24), MspJI (comprising also the distantly related Mrr) (25) and PvuRts1I families (26,27). The REases in this group tend to be two-domain proteins. The modification dependence is determined by a specificity domain, which is either of the SRA type (MspJI and PvuRts1I groups) (28,29) or a new fold (McrA group) (30). The McrA and MspJI specificity domains bind ^5m^C or ^5hm^C, but do not accept ^g5hm^C (25). In contrast, the PvuRts1I SRA domain depends on ^5hm^C or ^g5hm^C (26,27). For the McrA specificity domain, the mechanistic basis of modification dependence has not yet been determined. The SRA domains of MspJI, PvuRts1I and similar REases flip the modified nucleotide to scrutinize the DNA modification (31,32). Sequence specificity in addition to the modification dependence is present in some, but not all families. It has been inferred from DNA binding experiments for *E. coli* McrA (EcoKMcrA) (23) and is typical for the MspJI family members (33). In contrast, PvuRts1I and similar enzymes bind the modified cytosine irrespective of its sequence context (27).

The nuclease domains in the NTP independent, modification dependent REases are either of the HNH (McrA family) or PD-(D/E)XK type (MspJI and PvuRts1I families). Domain order varies. The nuclease domain comes first in some (PvuRts1I), but not other (McrA and MspJI) families (28,29,34). Sequence specificity of the nuclease domains has not been reported for any of the enzymes. The distance between sites of DNA modification and cleavage is accurately defined in the MspJI and PvuRts1I families (25,27), but variable for the McrA family (24,30). Therefore, the first two groups of enzymes are considered as Type IIM (recognition of a modified base and cleavage at fixed distance), whereas the McrA family is considered Type IV (recognition of a modified base and cleavage at variable distance) (35).

Recently, SRA-HNH REases have been added to the list of modification-dependent, remote site cleaving, NTP-independent DNA endonucleases (36). The SRA-HNH REases are characterized by the presence of an SRA (SET and RING finger associated) domain (37) at the N-terminus and an HNH (histidine-asparagine-histidine) domain (38) at the C-terminus. Two enzymes from the family, ScoA3IV from *Streptomyces coelicolor* A3 (Sco5333) and TbiR51I from *Thermobispora bispora* (Tbis1) have been studied experimentally. Both were found to be toxic to Dcm^+^*E. coli* hosts. In the test tube, the enzymes bind at least 100-fold better to DNA containing ^5m^C than to unmodified DNA, and exhibit weak DNA cleavage activity in the presence of Mg^2+^, Mn^2+^ and Co^2+^, but not Zn^2+^ ions (36).

Here, we present a biochemical and crystallographic characterization of a putative SRA-HNH endonuclease from the thermotolerant actinobacterium *Thermocrispum agreste*, termed TagI (35). Our data show that TagI is a member of the NTP-independent dimeric REases that cleave DNA at a distance from the modified cytosine base. TagI mediated DNA cleavage is enhanced by the presence of another modified base. The SRA domain of TagI accepts ^5m^C and ^5hm^C, but not ^g5hm^C. The TagI HNH domain is so far unique among the nuclease domains of modification dependent REases that cleave at a distance in exhibiting sequence specificity. The crystal structure of TagI explains the modification dependence, and preference for substrates with at least two modified bases. The properties of TagI do not fit conventional REase nomenclature and place it somewhere ‘in between’ Type IIM and Type IV enzymes.

## MATERIALS AND METHODS

### TagI gene cloning and purification

The synthetic *tagIR* gene with optimized *E. coli* codons (IDT) was cloned into pTXB1 (NdeI-XhoI cut) by NEB HI-FI assembly enzyme mix. The oligonucleotides used in this work including cloning primers are listed in Supplementary Table S1. The assembled DNA was transferred into Dcm^−^*E. coli* B strain C2566 by transformation (NEB). The same *tagIR* gene (a PCR fragment) was also cloned into pET28b (Novagen) to generate a C-terminal 6xHis-tagged version. The inserts were sequenced to confirm coding of the desired amino acid sequences. TagI production was carried out by IPTG induction overnight at 18°C. TagI was purified by chromatography through chitin (NEB) and Hi-Trap heparin (GE Healthcare) columns.

### Activity assays

#### Plasmid based assays

TagI restriction activity was first assessed by digestion of 0.5–1 μg of pBR322 (Dcm^+^ or Dcm^−^), pBRFM^+^ (Dcm^+^) or pACYC-HpyCH4IVM^+^ plasmid in NEB buffer 2.1 (50 mM NaCl, 10 mM Tris–HCl, 10 mM MgCl_2_, 100 μg/ml BSA, pH 7.9) at 37°C for 1 h. After the reaction Protease K (1.6 U) was added at 37°C for 15 min. The reactions were stopped by the addition of the RE stop buffer. The partially digested pBR322 was sequenced to map the cleavage sites. For the digestions carried out in the presence of Mn^2+^, 10 mM MgCl_2_ was replaced with 1 mM MnCl_2_. In the control experiments, 5 μg pBR322 was digested by 0.1 U of diluted DNase I in the DNase I buffer. Digestions were carried out at room temperature for 1, 2 and 5 min. The DNA was then subjected to spin column purification and sequenced. Control enzyme digestions (Fnu4HI, FokI, MspJI and MluCI) were carried out based on standard protocols.

#### Cleavage assay on modified PCR DNA

DNA fragments with ^5m^C or ^5hm^C (1.2 and 2.2 kb) were PCR amplified from pBR322 using Q5® DNA polymerase and ^5m^dCTP/^5hm^dCTP replacing dCTP. ^5hm^C-containing dNTPs were purchased from Zymo Research. ^5m^dCTP was from NEB. The cleavage assays carried out in the presence of Mg^2+^ were performed as above, i.e. in NEB buffer 2.1 at 37°C for 1 h. The comparison of TagI activity in the presence of various divalent metal ions was carried out in 0.1 M NaCl, 10 mM Tris–HCl (pH 7.5), 1 mM DTT, supplemented with divalent cations or EDTA.

#### Cleavage assay on modified oligonucleotides

For the comparison of digestion efficiency in the presence of one or several modified sites, we used four variants of the oligo_1 5′-ATG CAG AAC AAG CCG AAT TAA TAG ***GCGGC***C GAA GCT TAT AGC ATT GAT-3′ and four variants of the oligo_2 5′-ATC AAT GCT ATA AGC TTC G***GCCGC*** CTA TTA ATT CGG CTT GTT CTG CAT-3′ (***GCSGC***, Fnu4HI and TagI cut site) (IDT). The variants differed in the methylation status of the underlined cytosine bases. The oligonucleotides were separately annealed in all combinations to generate 16 different duplexes containing from none to four methylated cytosines. The digestion was carried out in the Mg^2+^ containing buffer at 37°C for 30 min. The reaction mixtures contained 1 μl (20 ng, 32 nM) oligoduplex, 1 μl TagI at 1/8 dilution (0.125 μg, 92 nM), 2 μl 10× NEB buffer 2.1 and 16 μl 10 mM Tris–HCl, pH 7.5. In the control digestions the same amount of oligoduplex was mixed with 20 U of Fnu4HI endonuclease (GC/NGC).

### TagI crystallization and data collection

Preliminary crystallization trials were performed in sitting drops using a Phoenix robot and the Morpheus and JCSG+ screening conditions. TagI protein (5 mg/ml) in the buffer containing 10 mM Tris–HCl, pH 7.5, 0.3 M NaCl, 5 mM β-mercaptoethanol (ME) and 0.5 mM EDTA, was mixed in 1:1 ratio with reservoir buffers. Preliminary screening identified 9 starting crystallization conditions. Since the crystals obtained in sitting drops proved extremely fragile and sensitive to manipulation, four conditions were selected for crystallization in 1 mm diameter glass capillaries. The best diffracting crystals were obtained by counter diffusion of 25 μl of the protein solution mixed with the 25 μl of 0.5% low melting agarose dissolved in the above buffer, and A5 Morpheus Screen (MDL) buffer (10% w/v PEG 20 000, 20% v/v PEG MME 550, 0.03 M MgCl_2_, 0.03 M CaCl_2_, 0.1 M MOPS/HEPES-Na, pH 7.5). Tetragonal crystals large enough to span the entire capillary diameter appeared after 2 weeks. The crystals had very high solvent content (70%) and were difficult to flash-cool. Therefore, a diffraction dataset was collected in house at room temperature on an X8 PROTEUM Bruker generator equipped with a MICROSTAR micro-focus X-ray source (Cu Kα radiation, 1.54 Å). The crystal was rotated approximately around the c-axis for quick completion of the dataset, which left the 0 0 l reflections in the dead cone region. Data were integrated and scaled using the SAINT and SADABS programs (Bruker, Inc). Together with extinctions on the h00 reciprocal space axis, the 4/mmm Laue symmetry identified the space group as *P*42(1)2, *P*4(1)2(1)2, *P*4(2)2(1)2 or *P*4(3)2(1)2. Data collection statistics are presented in Supplementary Table S2.

### Crystal structure determination

Models of the TagI SRA and HNH domains were built using the SWISSMODEL server (39), using the structures of the human UHRF1 SRA domain (PDB code: 3clz (40)) and the human ZRANB3 HNH domain (PDB: 5mkw (41)). The TagI structure was solved by molecular replacement using the PHASER program (42). Due to the much higher level of confidence in the model, the SRA domain was placed before the HNH domain. The SRA domain could also be oriented and positioned using the automatic BALBES server (43). Both protocols identified the space group as *P*4(1)2(1)2. The SWISSMODEL built HNH domain model (and alternatively also the original ZRANB3 fragment template) could then be placed using the FFFEAR program (44). The obtained composite models were submitted to iterative model building with the BUCCANEER (45) and ARP/wARP (46) programs which resulted in the 90% complete model and R and R_free_ of 21 and 27%. The model was refined using the COOT (47) and REFMAC (48) programs. The refinement statistics and quality indicators are presented in Supplementary Table S2. The atomic coordinates and the corresponding structure factors were deposited at the PDB with the 6GHS accession code.

## RESULTS

### Expression and purification of TagI endonuclease

BLAST searches of publicly available sequence databases using the well characterized SRA-HNH family REases, ScoA3IV and TbiR51I as queries, identify several hundred candidate endonucleases, most of them from *Actinobacteria, Bacteroidetes* and *Proteobacteria*, and rarely also from other bacterial phyla, including archaebacterial ones. We selected the putative SRA-HNH endonuclease from the thermotolerant, high GC-content, gram-positive *Thermocrispum agreste* for our studies. *T. agreste* grows well in a wide temperature range, between 28°C and 60°C (49). This suggested that the enzyme may have the stability benefits of proteins from thermophilic organisms, and yet be active at 37°C. TagI was recombinantly overexpressed from a codon-optimized synthetic gene in *E. coli*, as an intein-based self-cleaving chitin binding domain fusion protein. TagI expression was well tolerated in Dcm^−^ cells (T7 Express), but was toxic to Dcm^+^ cells, not only in the absence of RecA (NEB 5α, NEB 10β), but also in its presence (NEB Turbo) (Supplementary Figure S1). The enzyme was purified from whole cell extracts by chromatography on chitin and heparin columns. Except where otherwise indicated, all assays with the purified protein were carried out at 37°C.

### *In vitro* TagI dependence on metal cofactors and DNA modifications

The ^5hm^C-containing PCR DNA was efficiently digested by TagI in the presence of Mn^2+^, Co^2+^ and Ni^2+^ ions. The enzyme had lower activity in the presence of Mg^2+^ ions, and no detectable activity in the presence of Zn^2+^ or Ca^2+^ ions (Supplementary Figure S2). TagI activity was dependent on the modification status of the substrate. In Mg^2+^ containing buffer, TagI digested the Dcm methylated pBR322 and, at high enzyme concentrations, also cleaved the non-methylated plasmid (Supplementary Figures S3 and S4A). Digestion was also observed for pACYC-HpyCH4IVM^+^ with A^5m^CGT methylation (from a Dcm^−^ strain expressing M.HpyCH4IV), and was more efficient for pBRFM^+^ with additional G^5m^CNGC methylation (from a Dcm^+^ strain expressing M.Fnu4HI) than for only Dcm methylated pBR322. The data show that TagI can cleave substrates containing ^5m^C downstream of C (Dcm), G (M.Fnu4HI) or A (M.HpyCH4IV), and variously staggered methyl groups (Supplementary Figure S3). At high concentration, in high temperature or in Mn^2+^ conditions, TagI exhibited star activity and nicked/digested unmodified pBR322 (Supplementary Figure S4).

**Figure 1.**
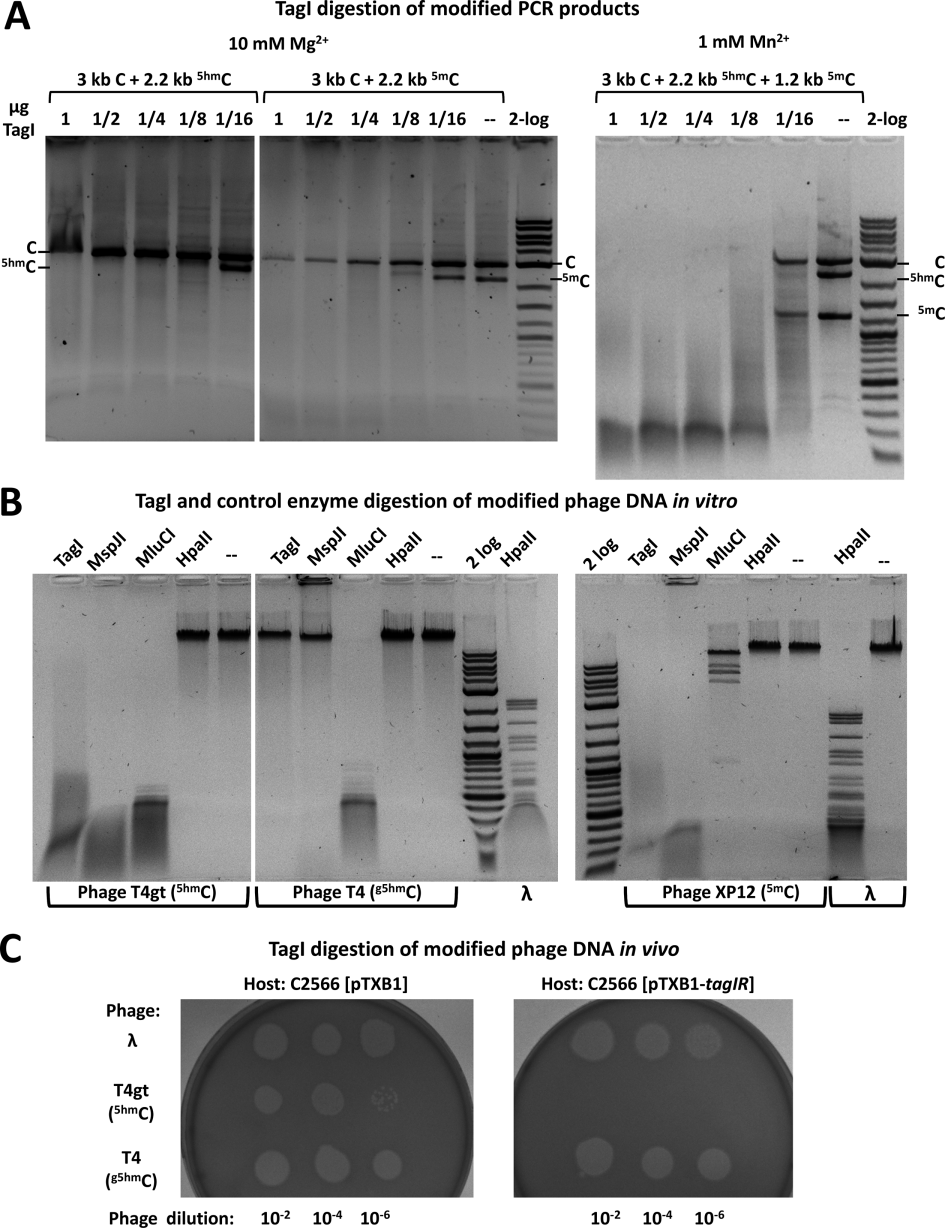
TagI activity assays on (A) ^5hm^C and ^5m^C containing PCR products and (B,C) modified phage DNA *in vitro* and *in vivo*. (**A**) One μg of mixed PCR DNA (∼12 nM) made from modified dNTP mixtures was digested at 37°C for 1 h with 1 μg TagI (∼0.3 μM) in 2-fold serial dilutions in NEB buffer 2.1. The substrates for TagI digestion in 10 mM Mg^2+^ contained C (3 kb) and ^5hm^C or ^5m^C (2.2 kb). The assay in 1 mM Mn^2+^ was performed on C (3 kb), ^5hm^C (2.2 kb) and ^5m^C (1.2 kb) containing PCR DNA. The amount of TagI (μg) shown on top of each lane corresponds to 295, 147, 74, 37, and 18 nM of protein dimer, respectively. (**B**) Modified DNA from phage T4gt (^5hm^C, ∼0.2 nM), T4 (^g5hm^C, ∼0.2 nM) or XP12 (^5m^C, 0.5 nM) was digested by TagI (∼0.3 μM) and control enzymes: tolerant to the presence of modified cytosines (MluCI (/AATT), 10 U), inhibited by cytosine modifications (HpaII (C/CGG), 10 U) and affected only by the presence of ^g5hm^C (MspJI, 5U). (**C**) Late-log phase host cells were plated on soft agar to form a cell lawn, and diluted phages (Lambda, T4gt or T4) were spotted onto the cell lawns. Cell lysis and plaque formation indicated susceptibility to phage infection. No plaque formation indicated the restriction of T4gt phage by TagI expressing cells.

The dependence of TagI activity on the type of cytosine modification was tested using PCR products made with either standard dNTP mix, or a mix containing either ^5m^dCTP or ^5hm^dCTP instead of dCTP. The PCR products had slightly different length, so that their fates in digestion reactions could be monitored independently. In the Mg^2+^ containing buffer, TagI cleaved the PCR products containing ^5m^C and ^5hm^C, but showed limited activity on unmodified DNA. In the presence of Mn^2+^ ions, the enzyme preferred the modified substrates, but in agreement with the results of the plasmid assays, in higher concentration also digested unmodified DNA (Figure 1A).

### Modification dependence of TagI activity tested on phage DNA

TagI activity on phage DNA was consistent with the assays on plasmids and PCR products. DNA containing either ^5hm^C (from glucosylation defective phage T4 (T4gt)), ^g5hm^C (from wt T4), or ^5m^C (from phage XP12) was subjected to cleavage by TagI and control restriction endonucleases (MspJI, MluCI and HpaII). The DNA from T4gt phage was digested by TagI, MspJI (^5m^CNNRN_9_/) and MluCI (/AATT), but not by HpaII (C/CGG, cleavage blocked by ^5hm^C modification) (Figure 1B). The DNA that was derived from intact T4 phage was resistant to MspJI and HpaII, but not to MluCI digestion, as expected. Interestingly, ^5hm^C glucosylation also blocked DNA cleavage by TagI. The ^5m^C containing DNA from XP12 phage behaved as expected, both in TagI and control digestions. The different outcome of TagI digestions of DNA containing ^5hm^C and ^g5hm^C was reproduced in phage plaque assays. The expression of TagI in *E. coli* cells had little effect on the outcome of infection by phage Lambda or T4. In contrast, the infection by phage T4gt was significantly attenuated (Figure 1C).

### Mapping of TagI cleavage sites

Run-off sequencing can be used to map cleavage sites in DNA. The method is based on the addition of an extra A by the sequencing Taq DNA polymerase to the nascent strand at the end of a template. A/C, A/G or A/T doublet or an unusually strong A indicates a nick in the template strand, on the 3′-side of the doublet with respect to the template, and the 5′-side with respect to the read strand. Alternatively, and equivalently, T/C, T/G, or T/A doublet or a strong T in the reverse complement point to a nick in the displayed strand, on the 3′-side of the ambiguous base (Supplementary Figure S5).

**Figure 2.**
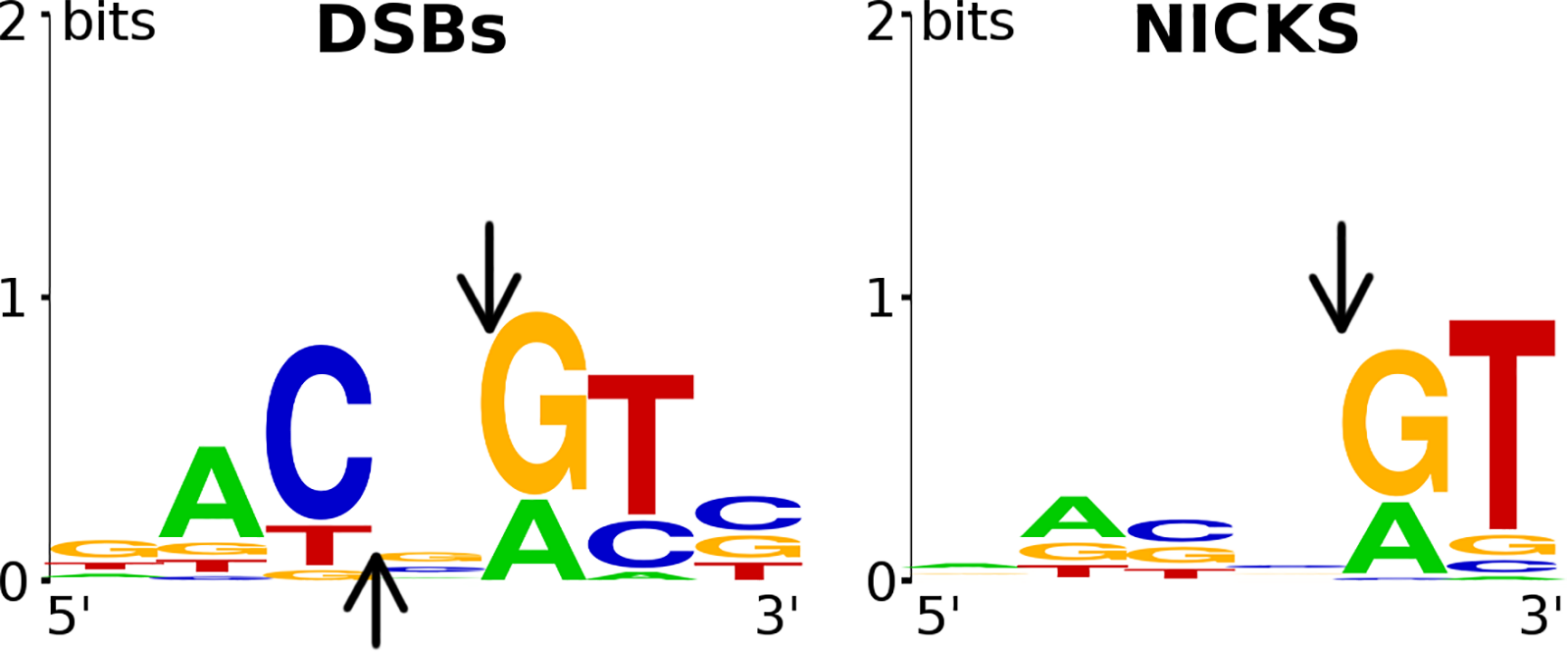
Sequence logo for TagI ds cleavage (left) or nicking (right) activity. 16 double strand cleavage sites (**A**) and 31 nicking sites (**B**) were combined from pBR322, pBRFM^+^, and ^5hm^C PCR DNA cleavage performed at 37°C. The arrow denotes the site of DNA cleavage or nicking. Note a systematic bias in the determination of the sequence logos. An A base immediately downstream of the cleavage site is detected with lower efficiency (because the polymerase incorporates the correct base, albeit in a template-independent manner).

TagI cleavage sites were mapped for pBR322 (C^5m^CWGG methylation) and pBRFM^+^ (G^5m^CNGC methylation) digestions carried out at 37°C and 50°C. Although the plasmids were only sparsely methylated (chance occurrence of C^5m^CWGG and G^5m^CNGC methylation sites for 50% GC content is 1:512 and 1:256, respectively), many doublets were found in close proximity of DNA modification sites (at a variable distance less than 20 2′-deoxynucleotides away). Nicking sites were detected in both strands in close vicinity to each other, suggesting that a concerted double strand break had occurred. In the majority of such cases, the nicks were staggered to generate single nucleotide 3′-overhangs in the digestion products (Supplementary Figure S5). The alignment of nicking sites indicated a preference for a purine–pyrimidine dinucleotide step (/RY) immediately downstream of the nick. The sequence for double strand cleavage was RYN/RY, as would be expected for single nucleotide staggered cuts and separate requirements to match the /RY consensus for both strands (Figure 2). At higher digestion temperature, suboptimal sites were more easily cleaved. Therefore, sequence logos indicated a less pronounced, but otherwise similar cleavage site preference (Supplementary Figure S5D).

Sequence preference only for the region downstream of a nick could be naturally explained by the polymerase preference (polymerase sees only one DNA strand, and only the region downstream of the nick). Therefore, we used the same protocol for run-off sequencing of control digestions performed using DNase I (non-specific endonuclease), MspJI and FokI (REases cleaving an undefined sequence at a specific distance from the recognition site). Neither the DNase I nor the MspJI and FokI controls exhibited the preferential nicking/cleavage on the 5′-side of a purine base (Supplementary Figure S6), confirming that the sequence logos for TagI DNA cleavage were not polymerase derived.

### TagI crystal structure

TagI was crystallized in capillaries by counter-diffusion. The crystals diffracted to 2.9 Å resolution, belonged to space group *P*4(1)2(1)2 and contained a single TagI protomer in the asymmetric unit. The structure was solved by molecular replacement and refined to typical quality indicators for this resolution (Supplementary Table S2).

**Figure 3.**
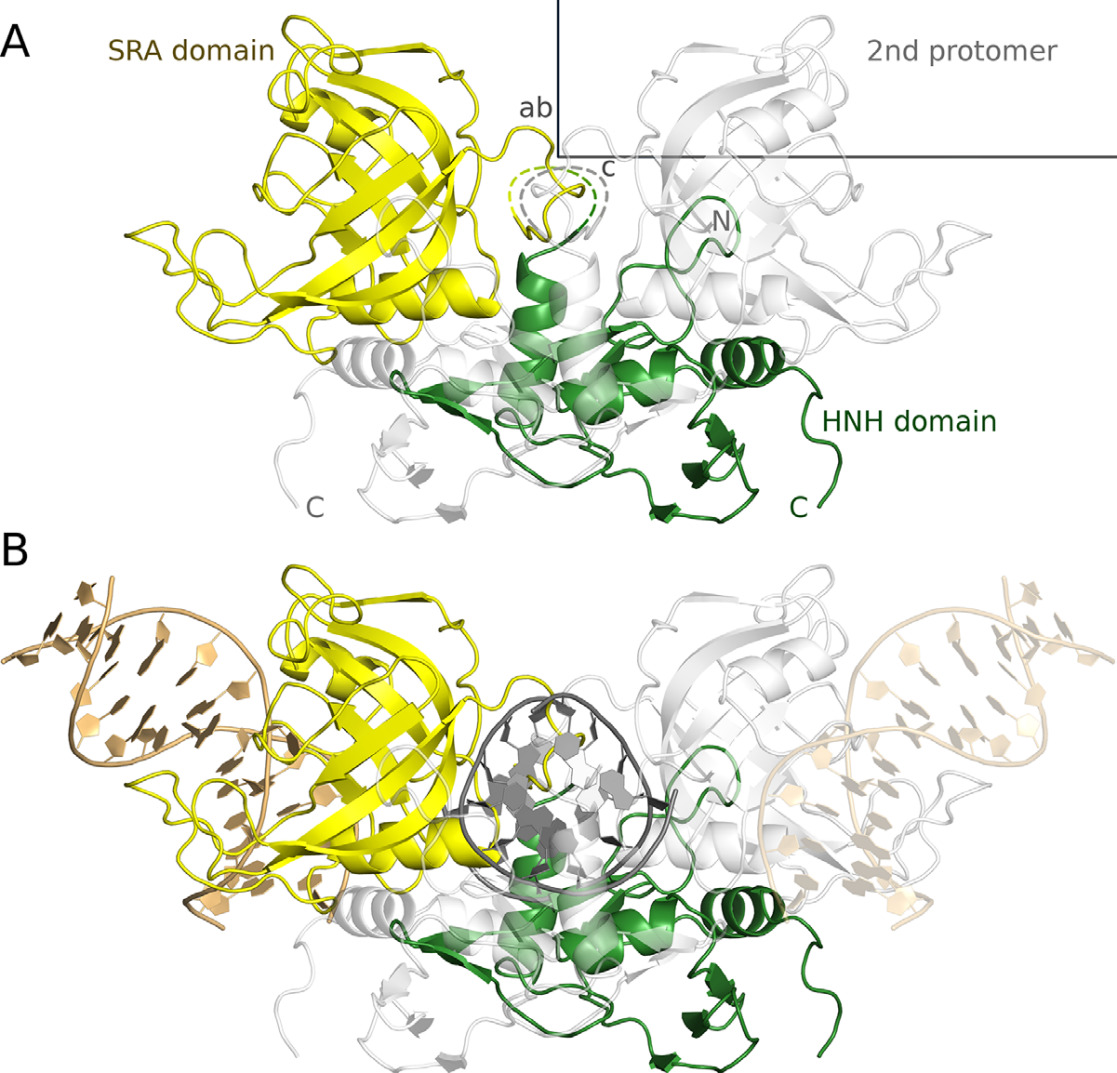
Experimentally determined structure of TagI in the absence of DNA (**A**) and a model of TagI with separate DNA fragments bound to the SRA and HNH domains (**B**). The TagI protomer in the asymmetric unit is shown in yellow (SRA domain) and green (HNH domain), the crystallographic symmetry mate that completes the TagI dimer is shown in light gray. The fragment of the structure that is disordered in the crystal is indicated by a dashed line. The unit cell and the directions of the crystallographic axes are shown in gray. The DNA molecules that have been modelled in complex with the SRA domains of the dimer are shown in gold and light gold color, and the single modelled DNA molecule that is bound to the HNH dimer is shown in dark grey color.

As predicted by the analysis of the amino acid sequence (36), TagI consists of an N-terminal SRA domain and a C-terminal HNH domain. The linker that connects the domains (residues 168–192) is not represented by electron density in the crystals, presumably due to disorder. This creates some uncertainties about the assignment of domains to polypeptide chains. We have assumed that the linker connects the closest C-terminal SRA and N-terminal HNH ends, however, the region of missing electron density would also be sufficient for an alternative assignment (Figure 3A).

Irrespective of how the domains are connected, it is likely that their packing against each other is determined by crystallization forces. The PISA web server (50) does not classify any interaction between the SRA and HNH domains (irrespective of assignment) as biologically relevant (|Δ^i^G| < 3.2 kcal/mol). This result suggests that the domains are mobile with respect to each other, in agreement with the biochemical result that TagI can cleave DNA at a variable distance from the modified DNA base.

TagI crystals contained a dimer located on a crystallographic two-fold axis and mediated by the interactions between the HNH domains. In contrast to the interaction between N- and C-terminal domains of the enzyme, the PISA web server (50) classifies the dimerization as biologically relevant. According to the PISA prediction, the interface area of the dimer is 1520 Å^2^ and the estimated free energy of dimer formation equals –12.8 kcal/mol. (Figure 3A).

### A model of TagI with DNA molecules bound to the SRA and HNH domains

The TagI SRA domain can be described, similarly to the prototypical UHRF1 SRA domain (26), as a distorted β barrel, which unlike perfect β-barrels has a ‘gap’, i.e. adjacent strands that should, but do not actually engage in main chain hydrogen bonding interactions. Thanks to the conservation of the dsDNA binding mode to the SRA domains (Supplementary Figure S7), the TagI SRA domain DNA interaction could be modelled with confidence. Among SRA domains of known structure, TagI is most similar to the human UHRF1 SRA domain, which has been crystallized by several groups in complex with dsDNA (1,40,51). The high sequence similarity between the two domains (42% amino acid identity over 194 residues), and conservation of key residues involved in DNA binding, support the model for the DNA complex of this domain (Figure 3B). Assuming flexible linkers between SRA and HNH domains, the two SRA domains may bind modified bases in opposite strands of the same DNA duplex. However, modelling suggests that this binding mode is dependent on the stagger of modified bases, and is possible for fully methylated ^5m^CG and ^5m^CNG, but prevented by clashes for fully methylated G^5m^C and GN^5m^C sequence contexts (Supplementary Figure S8).

The TagI HNH domain is built around the core ββα-Me motif of HNH endonucleases. The binding mode of DNA to the HNH domain of the enzyme was more difficult to model, due to the relatively low level of conservation between this domain and HNH domains that have been crystallized in complex with DNA. For the model, we took guidance from the structures of Hpy99I (52), I-PpoI (53) and T4 endonuclease VII (54). In all these protein complexes, the DNA is bent in a similar manner, with a widened minor groove, making it likely that the same distortion occurs also in the TagI DNA complex. The DNA bound to the TagI nuclease domains is likely canonically stacked, since the preferred target sequence with alternating purine and pyrimidine bases is not conducive to base stack rearrangements as in the PacI structure (55). Using the bent DNA from the Hpy99I complex, and the knowledge of the cleavage stagger (single nucleotide 3′-overhangs), we generated a model of the TagI HNH domain dimer with specifically bound DNA (Figure 3B).

The modelled DNA duplexes bound to the SRA and HNH domains do not align. Moreover, the regions of DNA bound to the two domains cannot be connected so that the distance between modification and cleavage site is only ∼8 nucleotides, i.e. the minimum distance that was found in the sequencing studies. The conclusion remains valid for alternative linker connections. We interpret this result as further evidence that the relative orientation of TagI SRA and HNH domains in the crystal may correspond to only one of many relative orientations that we expect to occur in solution.

### Predicted TagI SRA domain DNA interactions

**Figure 4.**
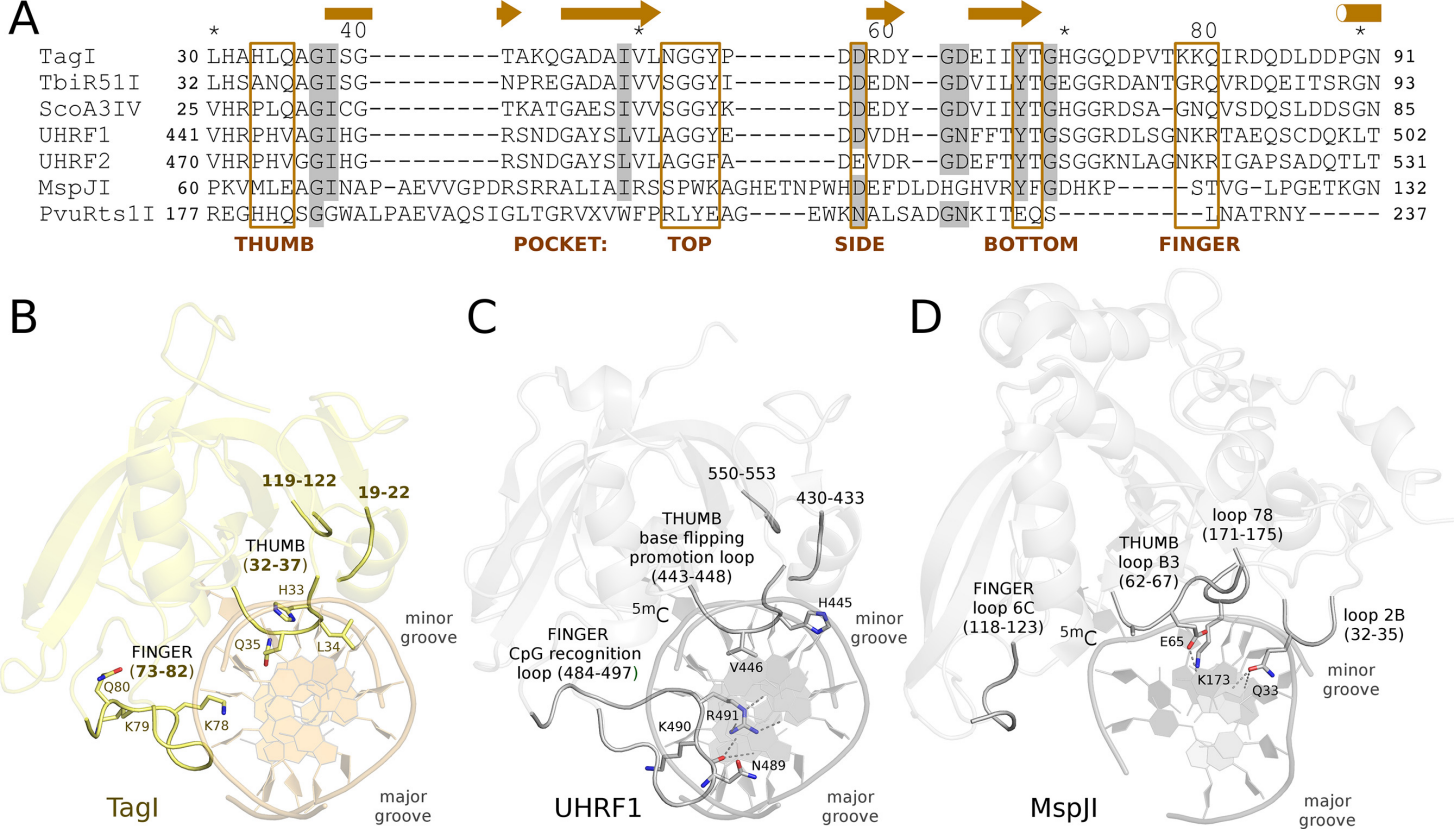
Sequence alignment (**A**) and structure of TagI (**B**), UHRF1 (40) (**C**) and MspJI (31) (**D**) SRA domains. The models are in ribbon representation. Key loops and selected functionally important residues are highlighted. The DNA in panel B is not present in the crystals and was modeled based on the UHRF1-DNA complex shown in panel C.

The interaction of the SRA domain with DNA resembles a hand grasping the DNA with ‘finger’ and ‘thumb’, both built from loops linking the strands of the β-sheet and the flanking α-helices (Figure 4 and Supplementary Figure S7).

The ‘finger’ of the SRA domain is also called the ‘NKR’ finger (40) or ‘CpG recognition loop’ (51) according to its amino acid sequence and functional role in the prototypical SRA domains of UHRF1 and UHRF2. However, the signature motif and role in CpG sequence specificity are not universal among the SRA domains. The equivalent loop in the MspJI group of enzymes is known as ‘loop 6C’ (31). Compared to UHRF1, the finger of the TagI SRA domain is much shorter and more compact. The equivalent of the NKR signature sequence of this loop in TagI is KKQ. The glutamine is not spatially equivalent to the arginine, which in the UHRF1-DNA complex interacts with the Hoogsteen edge of the estranged guanine (Figure 4BC).

The ‘thumb’ of the SRA domain is also known as the ‘base flipping promotion loop’ (51) because a residue anchored in this loop typically displaces the flipped base, or as ‘loop B3’ in the MspJI family of REases (28,33). The ‘thumb’ loop interacts with the DNA from the minor groove side. In TagI, Gln35 from this loop should fill the space of the flipped base, and appears well positioned to form two hydrogen bonds with the Watson–Crick edge of the estranged guanine base. In TagI, the thumb loop also anchors a histidine residue, His33, which could reach into the outer minor groove of the bound DNA or serve as a backbone binding residue.

Some SRA domains are specific for DNA sequence in the vicinity of the modified DNA base, e.g. 5′-^5m^CNNR-3′ for MspJI, 5′-YS^5m^CNS-3′ for AspBHI, and 5′-C^5m^CDG-3′ for LpnPI (33). The structural basis for this specificity is best understood in case of MspJI, which has been crystallized together with modified DNA. The specificity appears to be mediated by a favorable hydrogen bond of the so-called loop 78, which anchors Lys173. There is also a hydrogen bond from Gln33 to a universal hydrogen bond acceptor position in the DNA. The interacting residues are not conserved in TagI, and the corresponding loops are slightly further away in the TagI apo structure than in the MspJI-DNA complex. The conservation of the loops between TagI and UHRF1-DNA structures supports the conclusion about their conformation (Figure 4). The TagI model also predicts that a β-hairpin (approximately residues Gly37 to Val50 and a part of the distorted barrel) reaches towards the modelled DNA. The current model favors interactions of these residues with the DNA backbone over interactions with the minor grove edges of the DNA bases (Supplementary Figure S9). Nevertheless, we think that the model is not sufficiently accurate to exclude interactions that may mediate sequence specificity.

### Predicted TagI SRA domain pocket and specificity

**Figure 5.**
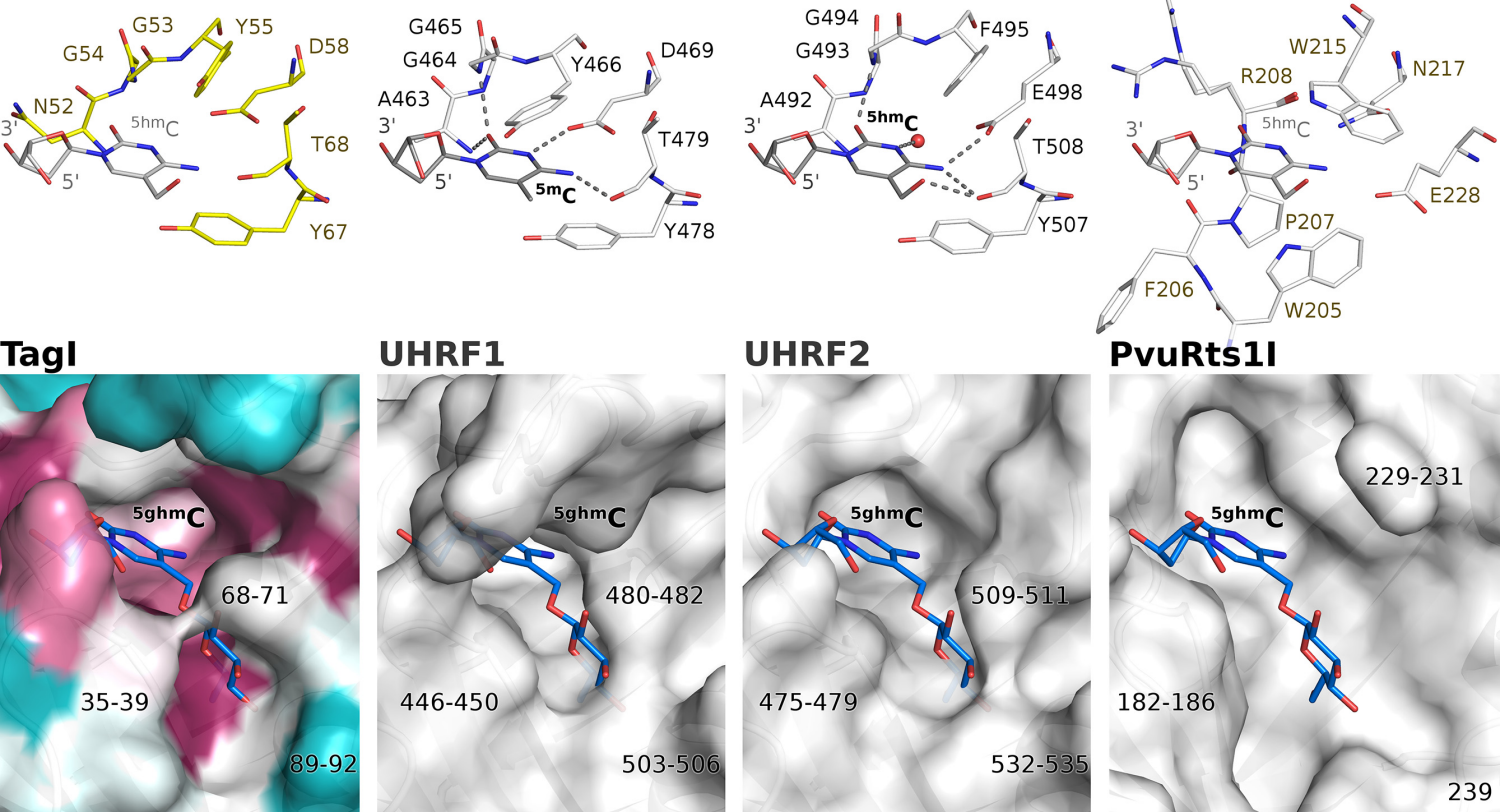
Specificity of the flipped base binding pockets in the SRA domains of TagI (**A**), UHRF1 (40) (**B**), UHRF2 (64) (**C**) and PvuRts1I (29) (**D**). Upper panels show the key residues forming the flipped base binding pockets. Lower panels depict the surface of the pockets. The TagI SRA domain surface was colored according to the sequence conservation calculated by the ConSurf server (57). The position of the flipped nucleotide in the TagI and PvuRts1I pockets is not based on experimental structure, but inferred from the binding mode of ^5hm^C to human UHRF2. The ^g5hm^C residue was modelled based on the NMR structure of β-d-glucosylated DNA (65). The binding modes of the ^5m^C in UHRF1 and ^5hm^C in UHRF2 are based on crystal structures.

SRA domains extrude the modified base from the base pair stack and bind it in a dedicated pocket. In the case of TagI (and in UHRF1), the walls of the pocket for the flipped base are built from tyrosine residues, Tyr55 and Tyr67. Here, the former tyrosine is rotated out from its position in the UHRF1 SRA domain, creating a slightly wider pocket similar to the one observed in UHRF2. As in other SRA domains, the Watson-Crick edge of the flipped base engages in several hydrogen bonds, with both main chain and side chain atoms. The most characteristic interaction is the hydrogen bond(s) with a carboxylate (of the aspartate in UHRF1 and glutamate in UHRF2). In the crystal structure of TagI, Asp58 is perfectly positioned to play this role (Figure 5, top). The interactions of the Watson–Crick edge of the base with the carboxylate are responsible for the preference for a modified cytosine over thymine. Assuming the same DNA backbone conformation, purines should also not be tolerated in this position because of their larger volume, which would lead to steric conflicts with the aspartate.

TagI cleaves DNA containing either ^5m^C or ^5hm^C, with a slight preference for ^5hm^C. The model shows that the SRA domain can accommodate both as well as an unmodified cytosine in the pocket. The preference for ^5m^C over C is presumably due to hydrophobic interactions and solvation effects, since the model places the methyl group in a fairly hydrophobic environment in the immediate vicinity of the side chains of Ile38 and Tyr67. The preference for DNA containing ^5hm^C could be due to formation of a hydrogen bond from the ^5hm^C hydroxyl group to the main chain carbonyl oxygen atom of Thr68. The threonine lies in a highly conserved region. Therefore, the explanation predicts a universal preference of SRA domains for ^5hm^C, in contrast to the experimental observations. We think that a ‘general’ SRA preference for ^5hm^C over ^5m^C is countered in some, but not all SRA domains by the conformation of the first of the two aromatic residues (Tyr55 in TagI, Tyr466 in human UHRF1 and Phe495 in human UHRF2). It can adopt an ‘in’ conformation as in UHRF1 that collides with ^5hm^C, or an ‘out’ conformation as in UHRF2 or TagI that leaves space for the hydroxyl group. The choice between ‘in’ and ‘out’ conformation appears to depend on the local environment, and not on whether the residue is phenylalanine or tyrosine (Figure 5, top).

TagI does not cleave DNA containing ^g5hm^C, in contrast to enzymes of the PvuRts1I family, which accept and even prefer the presence of ^5hm^C glucosylation. Modelling indicates that a glucosyl group would clash with TagI in the region of Thr68-Gly71 residues. This region adopts a similar conformation in UHRF1, but differs substantially in PvuRts1I, which has enough space for the glucosyl group. Further regions that may be involved in the ^g5hm^C discrimination comprise residues 35–39 and 89–92 of TagI. The first region adopts slightly more favorable conformation in PvuRts1I and the second is almost completely missing. In summary, we predict that the lack of activity of TagI towards ^g5hm^C is at least in part caused by the SRA domain, but we of course cannot exclude that the HNH domain may separately reject DNA with this modification (Figure 5, bottom).

### TagI HNH domain

**Figure 6.**
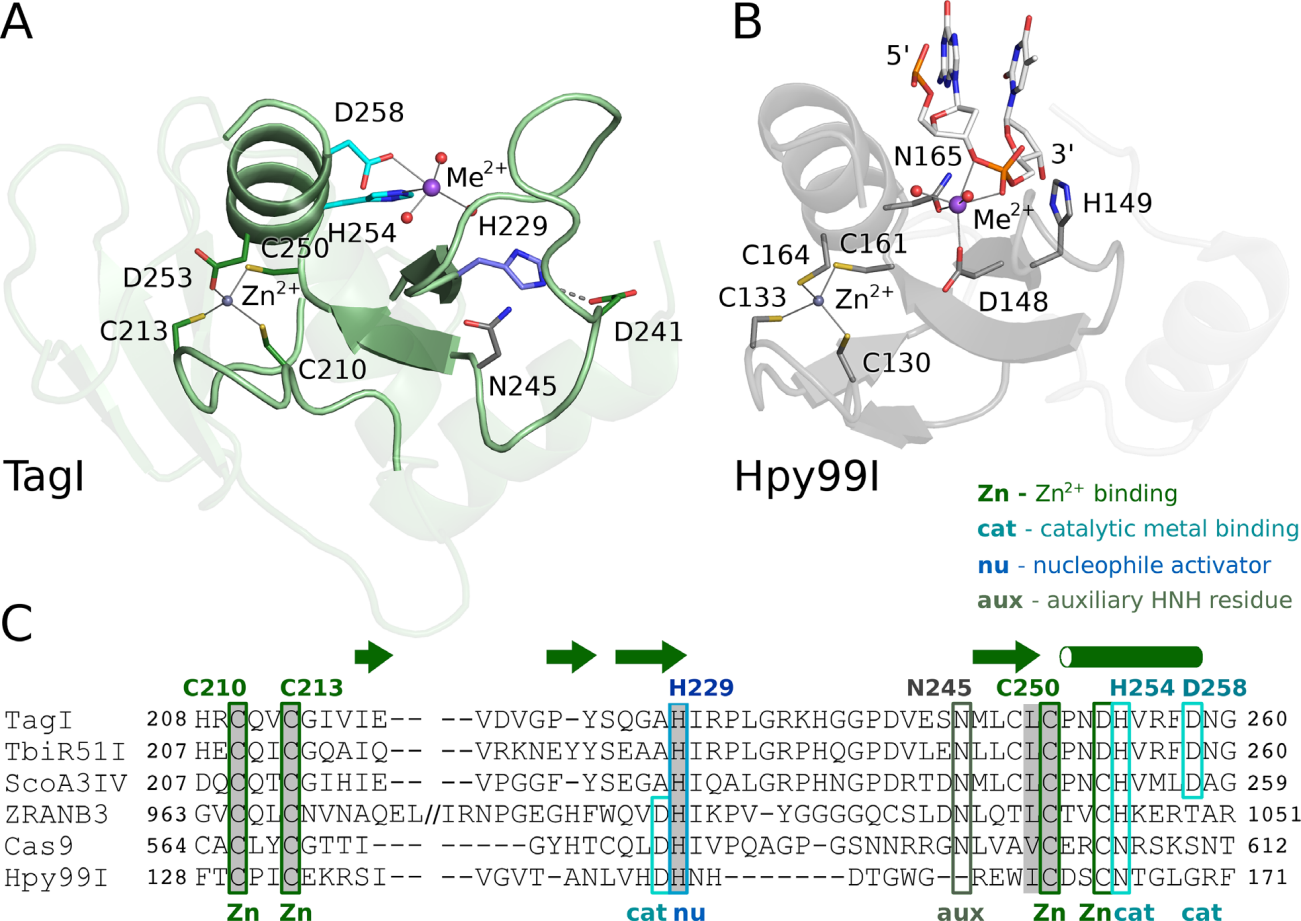
HNH domain of TagI (**A**) and of the Hpy99I REase with bound DNA (52) (**B**), and sequence alignment of selected HNH nucleases (**C**). The ββα-Me motif that is characteristic for the HNH nucleases is highlighted by more intense coloring. Selected active site residues and the ligands of the structural Zn^2+^ ion are indicated. In panel (B), only the dinucleotide around the scissile phosphodiester bond is shown.

The TagI HNH domain is organized around two divalent metal cations, a structural Zn^2+^ ion and a catalytic divalent metal ion (Figure 6). The identity of the latter ion in the crystal is uncertain, but we tentatively interpret it as a Na^+^ ion. Mg^2+^ or Ca^2+^ ions were also present in the crystallization buffer, but their concentration was much lower than for EDTA, and thus they were most likely chelated and unavailable for the enzyme.

The structural Zn^2+^ ion in TagI is coordinated by three cysteines, presumably in the thiolate form, and an aspartate (Figure 6). The sequential spacing between the metal chelating residues is typical for the Zn^2+^ binding motif in HNH endonucleases and many other Zn^2+^ chelating proteins in general (41,52,56). The Cys210 and Cys213 residues of TagI form a classical CxxC motif. Replacement of either of the two cysteine residues by an alanine drastically reduced the activity, so that at the highest tested concentrations, at most nicking or linearization of a Dcm methylated pBR322 plasmid were observed (Supplementary Figures S10 and S11). TagI Cys250 and Asp253 represent a CxxD variant of the Zn^2+^ binding sequence, that requires only slight backbone changes compared to the classical CxxC motif, since the cysteine and aspartate side chains have similar lengths. The TagI CxxD motif is not characteristic for the entire family of SRA-HNH endonucleases. Other family members have either a conventional CxxC motif (e.g. ScoA3IV) or a functionally equivalent CxxH motif (e.g. CflDI putative endonuclease, GeneBank code: ADG74130.1) (Figure 6C) (36).

The catalytic divalent metal ion, or the surrogate Na^2+^ ion in the crystals, defines the location of the active site, which is anchored in the typical ββα motif that has given rise to the alternative ββα-Me designation for HNH endonucleases (Me stands for the metal ion). TagI is a ‘canonical’ HNH endonuclease, because the moniker histidine, asparagine and histidine residues are present as suggested by the nuclease family designation and not replaced by functionally equivalent residues (Figure 6).

The first histidine of the HNH motif, His229 in TagI, is typically considered as the residue that activates the nucleophilic water for the phosphor-ester scission (52,53). In the TagI structure, this histidine is held in place by a hydrogen bond (or a salt bridge) to Asp241. The nucleophile activating His229 of TagI, is found in the secondary structure context typical for HNH endonucleases, i.e. at the end of the first β-strand (residues 226–229) of the ββα-Me motif. The asparagine of the HNH motif is frequently conserved, but has no catalytic role (52). Asn245, the corresponding residue in TagI, is hydrogen bonded with main chain of Arg231 and may help to stabilize the catalytic core motif. The second His residue of the HNH motif, His254 in TagI, is involved in the coordination of the active site metal. All three residues are essential, as judged from sequence conservation and site-directed mutagenesis (Figure 6, Supplementary Figures S10 and S11, Supplementary Table S3).

Additional coordination of the metal ion is interesting. In many HNH endonucleases, an acidic residue directly upstream of the first histidine of the HNH motif is a first shell ligand of the active site metal ion. However, Ala228 in TagI is not suitable for this role. Instead, an acidic residue, Asp258, located three amino acids downstream of the last histidine of the HNH motif, appears to take this function. An aspartate residue in this position is present also in several other SRA-HNH endonucleases, including the prototypical ScoA3IV, but not all enzymes in the family (36). The activity assay confirms that the TagI aspartate is required for activity (Figure 6C, Supplementary Figures S10 and S11).

Prior to the structure determination of TagI, we also prepared H237A, N259A and H288A variants of the enzyme. His237 is located close to the HNH dimer interface, ∼11 Å away from the expected position of the scissile phosphate. When the residue was replaced by alanine, more activity was retained than in the case of the active site mutants (Supplementary Figure S10). The N259A and H288A variants had only very weak activity, and were at most able to nick or linearize the test substrate. Asn259 engages in hydrogen bond formation at the dimer interface and lies immediately downstream of Asp258, one of the residues that bind the catalytic metal ion. Therefore, the importance of Asn259 is not surprising. His288 is located at the surface of TagI, away from the active site, predicted DNA binding region and dimerization interface. The strong effect of an exchange of His288 is therefore difficult to explain (Supplementary Figure S11).

### Predicted TagI HNH domain DNA interactions

The RYN/RY consensus sequence around TagI cleavage sites lies within in the estimated footprint of the HNH dimer. Therefore, the specificity for the bases in the immediate proximity of the cleavage site must stem from interactions of the HNH domains with the DNA. Unfortunately, the TagI HNH-DNA model is less confident than that of the SRA domain and does not make it possible to predict detailed interactions. Nevertheless, some general conclusions can be drawn. In the model, there are no clear contacts between TagI and the central base pair of the recognition sequence. However, several TagI residues, all with a possibility to engage in sequence selective hydrogen bonding interactions, come close to the other bases of the recognition sequence.

Arg194, which inserts into the major groove of the bound DNA in the model, makes contact with the +1 purine of the recognition sequence (i.e. the R immediately downstream of the central nucleotide). However, inaccuracies in the model make it also possible that Arg194 may interact with the base pairs in ±2 positions. Moreover, this residue is at the end of the ordered part of the inter-domain linker that is likely to adopt different conformation in the DNA complex. Gln226, the residue that forms hydrogen bonding interaction with His254 coordinating the active site metal, approaches the DNA from the minor groove side. Lys129 and a carbonyl oxygen atom of the linker could also come close to the DNA. Finally, it is possible that the linker may become ordered in the catalytic complex and may contribute additional sequence specific interactions upon DNA binding.

### TagI oligomerization and DNA binding in solution

In order to check the oligomeric state of TagI in solution, we carried out analytical gel filtration experiments, either for TagI alone, or in the presence of unmodified, hemi- or fully-methylated 17mer DNA duplexes (modified in the ^5m^CNG context) (Supplementary Figures S12 and S13). TagI alone migrated with an apparent molecular mass of 68 ± 3 kDa, close to the theoretical 67.9 kDa mass of the protein dimer (Supplementary Figure S14). Multi-angle light scattering (MALS) confirmed the molecular mass of 68 ± 3 kDa, in excellent agreement with expectation (Supplementary Figure S15). As foreseen, oligoduplexes alone migrated faster than expected due to the elongated rod shape (at the apparent mass of 22 kDa in contrast to the calculated mass of 10 kDa). When TagI and unmodified DNA were co-injected, they migrated independently in any tested stoichiometric ratio (1, 2 or 4 DNA duplexes per TagI dimer). Co-injection of TagI and hemi-methylated DNA (either strand methylated) led to a broad peak for complex and to a sharper peak for DNA alone (when DNA was present in excess). The data suggest that there was equilibrium between unbound TagI and the protein bound to hemi-methylated DNA. In contrast, co-injection of TagI and fully methylated DNA resulted in sharp peaks for the complex (apparent mass of 93 kDa) and unbound DNA (when DNA was present in excess). We conclude from these data that fully methylated DNA bound more tightly to TagI than hemi-methylated DNA. At most slightly more than one duplex of DNA per TagI dimer co-migrated, even when DNA was present in several-fold excess. The tighter binding of fully methylated DNA and the unexpected saturation already at one DNA duplex per TagI dimer indicated that one SRA domain could be bound to each DNA strand of the duplex. This interpretation was additionally supported by the presence of a small peak at still lower retention time (corresponding to the apparent mass of ∼220 kDa), which was most likely due to two TagI dimers bound to a single DNA duplex (or other super-complexes). The interpretation that two SRA domains of a TagI dimer could be bound to two methyl groups of a fully methylated DNA duplex was in agreement with the modelling result that two SRA domains could be placed on fully methylated ^5m^CNG DNA without serious clashes (Supplementary Figure S8).

### Guidance of TagI by one or two DNA modifications

**Figure 7.**
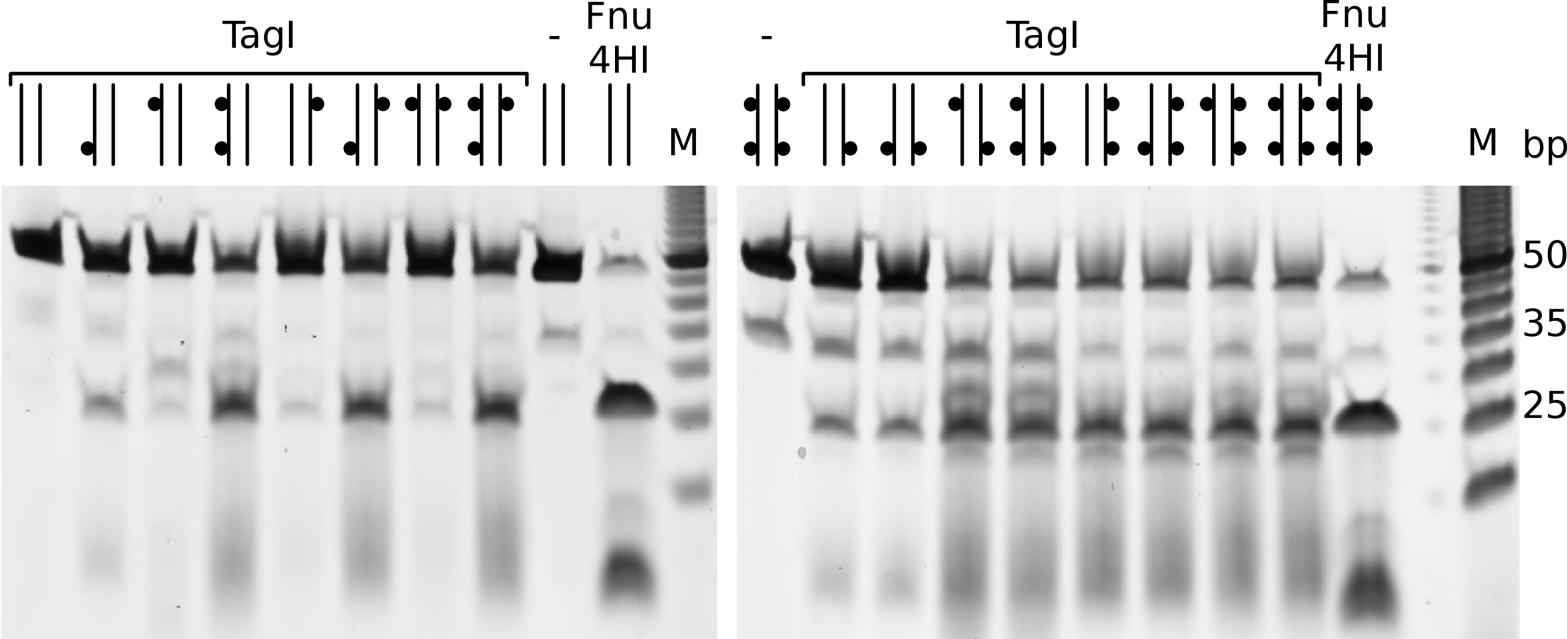
Efficacy of DNA TagI cleavage of substrates containing none to four ^5m^C bases. 20 ng (∼32 nM) of annealed 48-mer oligonucleotides were digested by TagI (0.125 μg, ∼92 nM of TagI dimer) in NEB buffer 2.1 for 30 min. Cleavage products were resolved in 15% urea-PAGE, stained by SYBR Gold and imaged on Typhoon imager. The predicted TagI cleavage site GCS/GC at the center of the duplexes conforms to the GC/NGC consensus for cleavage by Fnu4HI (20 U), which was used as a positive control. ^5m^Cs in oligoduplexes are represented by small black dots in the diagrams above each lane.

The architecture of TagI with two SRA domains that separately bind duplex DNA, but only a central (dimeric) HNH nuclease, suggested that the enzyme may cleave DNA more efficiently when guided by two instead of a single modified base. In order to test this hypothesis, we compared the cleavage of oligoduplexes that had a GCSGC site compatible with the TagI RYN/RY nuclease specificity, flanked by either unmodified or methylated cytosines in top and bottom strands separated from the cleavage site by 11 or 12 nucleotide spacers (5′-N_11_-GCC-N_10_-GCGGC-N_11_-GCA-N_5_-3′ and 5′-N_6_-GCT-N_10_-GCCGC-N_11_-GCT-N_10_-3′). In this way, all 2^4^ = 16 combinations of methylation at the four sites could be separately probed. The digestions were carried out in the presence of Mg^2+^ ions to keep the reaction rates relatively low and to concentrate cleavage at a consensus GCG/GC sequence between the two modification sites (Figure 7). Under these conditions, the control oligoduplex without DNA modifications was not cleaved at all.

Oligoduplexes containing only a single ^5m^C were hydrolyzed slowly. Interestingly, a single ^5m^C could direct cleavage either upstream or downstream of the modified base. Oligoduplexes containing at least two ^5m^Cs could be divided into two groups. Those that had two neighboring ^5m^Cs in top and bottom DNA strand were cut inefficiently, like the ones containing only a single ^5m^C, presumably because the sequence context was (arbitrarily) chosen as G^5m^C, which should not be compatible with simultaneous binding of two SRA domains to a fully methylated site (Supplementary Figure S8). We expect that for ^5m^CG and ^5m^CNG sequence contexts, such oligoduplexes should be efficiently cleaved. All duplexes with ^5m^C modifications at a distance (28 bp in our experimental conditions) were hydrolyzed efficiently. For such substrates, cleavage was effective for symmetry compatible modifications on opposite strands and symmetry breaking modifications in the same strand. The enhancement rate appeared to be more than two-fold compared to substrates with a single modified C, and therefore was unlikely to result from twice more efficient nuclease recruitment alone.

Oligoduplexes containing three or more ^5m^C sites necessarily contained at least one pair of ^5m^C sites in mutually supportive positions. As expected based on the results for the duplexes with exactly two distant ^5m^Cs, all were cleaved efficiently. We conclude that TagI works optimally under the guidance of two modified cytosine bases flanking a consensus cleavage site, irrespective of which strand the methyl groups are placed in (Figure 7).

### An unbiased screen for inactivating TagI modifications

TagI is toxic to Dcm^+^ cells, in both RecA^+^ and RecA^−^ (DNA repair deficient) background. We used error-prone PCR with Mn^2+^ instead of Mg^2+^ ions to introduce mutations in the TagI gene, and scored the resulting expression constructs for toxicity to *E. coli* cells. Plasmids expressing ‘non-toxic’ TagI were then sequenced (Supplementary Table S3). As cells can repair nicks more easily than double strand breaks, it was expected that some TagI variants could retain nicking activity. It was indeed found that some of them clearly retained the property. Variants were surprisingly evenly distributed across the entire TagI enzyme, and did not cluster at the active sites or predicted DNA binding regions. For the relatively small number of characterized variants, there was also no clear correlation with conservation scores calculated using the Consurf server (57) (Supplementary Figure S16).

## DISCUSSION

### A model for TagI activity

Our combined data suggest a simple model for TagI activity. We attribute the modification specificity to the SRA domains, and the RYN/RY specificity to the HNH-domain dimer. We expect that the TagI SRA domains anchor the enzyme to sites of DNA modification. Whether they exhibit sequence specificity like many better-characterized SRA domains (1,33,40,51) remains currently unknown. However, the *in vitro* activity on DNA that has been methylated by Dcm (C^5m^CWGG), M.Fnu4HI (G^5m^CNGC) or M.HpyCH4IV (A^5m^CGT), suggests that the TagI SRA domain can bind to DNA that is modified in various sequence contexts. For physiologically relevant (low) TagI concentration, we expect that cleavage occurs only when the nuclease domains encounter a target site and are co-anchored to this site by one or two DNA modifications. As the TagI dimer has a single DNA binding site in the nuclease domains, but two separate binding sites for modified cytosines in the SRA domains, it is unsurprising that two suitably spaced modified bases can direct DNA cleavage at a single recognition sequence more efficiently than a single modified base. The guidance from two modification sites may also explain the efficient cleavage of heavily modified PCR DNA or phage DNA such as XP12 and T4gt.

Our model of TagI activity is not readily compatible with the standard classification of REases (35). The enzyme can be described as a Type IV REase, because it cleaves DNA at a distance from a site of modification. However, the enzyme also exhibits features of a standard Type II REase with defined target sequence, although semi- or full degeneracy in every single position are not typical. TagI cannot be described as a Type IIM REase, because the definition of such enzyme requires that the modification must be present within the target sequence.

### Properties of modular, NTP-independent, modification dependent REases

The modular architecture of TagI is widely shared among the NTP-independent, modification-dependent endonucleases. TagI shares an HNH domain as the nuclease domain with the EcoKMcrA (MCRAN-HNH) family (34,30), and the SRA domain with the PvuRts1I (PD-(D/E)XK–SRA) (29,58,59) and MspJI/Mrr (SRA–PD-(D/E)XK) (28,31) families.

The non-catalytic domains in these fusion proteins bind DNA in modification dependent manner. With its requirement for ^5m^C or ^5hm^C, but not ^5ghm^C, TagI exhibits similar modification dependence as the EcoKMcrA (30) and MspJI/Mrr families (25), but not the PvuRts1I family (26,27). Thus, modification specificity does not segregate according to phylogeny. Typically, but not universally, the modification-dependent, non-catalytic domains have some sequence specificity (in the EcoKMcrA and MspJI/Mrr families, but not in the PvuRts1I family) (27,33,30). The sequence specificity of the non-catalytic TagI domains is not yet fully clear, but we know already that the enzyme can cleave DNA which is methylated in C^5m^CWGG, G^5m^CNGC and A^5m^CGT sequence contexts (the C opposite to the underlined G is also modified), suggesting that the TagI SRA domain has broad sequence specificity (Table 1).

**Table 1.** Properties of modification dependent REase groups. The modification dependent REases may be classified according to their domain organization. The three best known groups of modified cytosine dependent REases are characterized by similar modification, distance and sequence preferences

	Modification dependent domain		Interdomain	Nuclease domain	Multiple modifications	
REase family	sequence specificity	modification specificity	modification-cut distance range	sequence specificity	cleavage enhancement	symmetry requirement
**SRA - HNH**	**unknown**	**^5m^C, ^5hm^C**	**broad**	**YES**	**YES**	**NO**
TagI	unknown	^5m^C, ^5hm^C	9-23	RYN/RY	YES	NO
**McrAN - HNH**	**YES**	**^5m^C, ^5hm^C**	**broad**	**broad**	**NO**	**NO**
EcoKMcrA	(Y)^5m^CGR	^5m^C, ^5hm^C	broad	broad	NO^a^	NO
**SRA - PD-(D/E)XK**	**YES**	**^5m^C, ^5hm^C**	**defined**	**broad**	**NO**	**NO**
MspJI	^5m^CNNR	^5m^C, ^5hm^C	12	broad	NO	NO
AspBHI	YS^5m^CNS	^5m^C, ^5hm^C	10	broad	NO	NO
LpnPI	C^5m^CDG	^5m^C, ^5hm^C	12	broad	NO	NO
**PD-(D/E)XK - SRA**	**NO**	**^5hm^C, ^g5hm^C**	**narrow**	**broad**	**YES**	**YES**
PvuRts1I	NO	^5hm^C, ^g5hm^C	11-13	broad	YES	YES
AbasI	NO	^5hm^C, ^g5hm^C	11-13	broad	YES	YES

^a^The enhancement of EcoKMcrA activity by multiple modification sites is not observed *in vitro*. However, the activity of the enzyme in the test tube is low and it remains possible that its natural substrate has not yet been identified.

The catalytic domains of the modular, modification dependent and NTP-independent REases are generally assumed to be sequence non-specific. TagI is unusual in this respect, because the nuclease domains exhibit clear sequence preference, both for nicking and double strand breaks. In the small sample of modification dependent REases studied to date, fixed distances between modification and cleavage sites, and nuclease sequence preferences appear anti-correlated. This may not be accidental, since both bring down the number of cleavage sites, perhaps to balance toxicity for the host with efficacy against invading DNA (Table 1).

In all families of modification dependent REases discussed here, the nuclease domains dimerize, but the non-catalytic domains do not (28–30). As a result, the nuclease domains form a joint DNA binding site, whereas the non-catalytic domains expose separate sites. The architecture suggests that DNA double strand breaks can be controlled by up to two modified bases. The MspJI family is generally considered to require only a single modified base (25), and this may also apply to the EcoKMcrA REases, for which the physiological substrate is still uncertain (30). In contrast, the PvuRts1I family operates optimally when two suitably spaced modified DNA bases direct DNA cleavage (27). However, the requirement for two sites is not rigorous, otherwise the enzyme could not be used to map ^5hm^C in eukaryotic DNA (26,27,60), despite the rarity of the modified base (61,62). With respect to the number of modified bases required for optimal cleavage, TagI is thus most similar to the PvuRts1I family in being aided by, but not dependent on two modified DNA bases. At least in the canonical PvuRts1I substrates, modifications have to be symmetrically arranged (on the opposite DNA strands) (27). This is not the case for TagI, which cleaves substrates with two modified bases in the same strand with similar efficiency as substrates with modifications on opposite strands.

### Biological implications

In view of the likely limited selectivity of TagI for the sequence context of the modified base, it appears surprising that it is not toxic to the host. The potential conflict between C5-methyltransferases and TagI appears to be avoided by the absence of the former. The BLASTP or TBLASTN (63) search of the *T. agreste* proteome or genome, using the *E. coli* Dcm protein sequence as a query, did not identify hits that are likely to encode DNA methyltransferases. Therefore, it is plausible that *T. agreste* lacks Dcm methylation, and perhaps all C5 genomic methylation, and can thus tolerate the TagI activity. In the *Streptomyces coelicolor* A3(2) genome, where the prototype SRA-HNH endonuclease ScoA3IV was first discovered, there is one putative C5 methyltransferase gene (ScoA3ORF6844). *S. coelicolor* A3(2) carries three ^5m^C-dependent restriction systems, namely ScoA3I (unverified, SauUSI-like), ScoA3IV, and ScoMcrA (36). It is therefore likely that ScoA3ORF6844 is not an active DNA methyltransferase, which would be consistent with the presence of an SPPC instead of the canonical GPPC active site motif (motif IV) in its sequence.

The ^5hm^C and ^g5hm^C bases are typically only found in phage DNA. ^5hm^C is synthesized by the phage from a mix of dNTPs, with ^5hm^dCTP instead of dCTP. The modification of cytosine happens at the level of the monophosphate, and is catalyzed by a deoxycytidylate 5-hydroxymethyltransferase (dCMP HMase) with similarity to folate dependent thymidylate synthases (6). Using the T2 phage dCMP HMase as the query, we confirmed the absence of similar proteins from the *T. agreste* proteome, thus excluding the possibility of ^5hm^C production from an integrated prophage.

It is currently somewhat unclear what exactly TagI defends its host against. *T. agreste*, the producer organism of TagI, belongs to the *Actinomycetales*, an order of *Actinobacteria*. BLAST searches using TagI as a query confirm that this origin is typical, and that many other SRA-HNH endonucleases are found in *Actinobacteria, Bacteroidetes* and *Proteobacteria*. Abundant d^5m^CMP instead of dCMP may be present in *Achromobacter, Roseobacter*, and *Xanthomonas* phages (6), that are known to infect *Proteobacteria* only. Enzymatic machinery to generate the ^5hm^CTP building block is present in T4, T4-like, *Enterobacteria, Xanthomonas, Aeromonas* and *Salmonella* phages that also infect *Proteobacteria*. ^g5hm^C has been detected in the genomes of various phages. Non-glucosylated ^5hm^C has so far not been found in phage genomes, but genomic data suggest that some phages may have enzymes to generate ^5hm^C and may lack the activity to glucosylate it (6).

The taxonomic mismatch between the origin of TagI and many related SRA-HNH endonucleases on the one hand, and the origin of phages with hypermodified genomes containing either ^5m^C or ^5hm^C on the other hand, is surprising. It may suggest that TagI is primarily directed against sparsely C5-methylated phage genomes, which could have arisen by propagation in hosts with active C5-methyltransferases. Alternatively, phages with hypermodified genomes may have a broader host range than expected. Finally, the data may also be a hint that novel phages with modified genomes that could be targets for TagI and other SRA-HNH endonucleases, remain to be discovered.

## DATA AVAILABILITY

The atomic coordinates and the corresponding structure factors have been deposited at the PDB with the 6GHS accession code.

## Supplementary Material

Supplementary Data
