# Supplementary material for: Crystal structure of the modification-dependent SRA-HNH endonuclease TagI
Source: Nucleic Acids Res. 2018 Sep 7;46(19):10489–503. doi: 10.1093/nar/gky781 (PMC6212794; doi:10.1093/nar/gky781)
Supplement: Supplementary Data [file gky781_supplemental_files.pdf]

Supplementary Material for

Crystal structure of the modification-dependent SRA-HNH endonuclease TagI

Marlena Kisiala[1,2,3], Alyssa Copelas[4], Honorata Czapinska[1],
Shuang-yong Xu[4*], Matthias Bochtler[1,2*]

[1]International Institute of Molecular and Cell Biology, Trojdena 4, 02-109 Warsaw, Poland
[2]Institute of Biochemistry and Biophysics PAS, Pawinskiego 5a, 02-106 Warsaw, Poland
[3]Biological and Chemical Research Centre, University of Warsaw, Żwirki i Wigury 101, 02-089 Warsaw, Poland
[4]New England Biolabs, Inc. 240 County Road, Ipswich, MA 01938, USA

*Co-corresponding authors:

Shuang-yong Xu, xus@neb.com
Matthias Bochtler, mbochtler@iimcb.gov.pl

Supplementary Methods

Site-directed mutagenesis and random mutagenesis by error-prone PCR

Site-directed mutagenesis was carried out according to the Q5® Site-Directed Mutagenesis Kit protocol, using primers from IDT, and pTXB1-*tagIR* as template. After PCR, the mix was treated with DpnI (to destroy the wild-type (WT) template), T4 polynucleotide kinase and DNA ligase. The mutagenized DNA was transferred into C2566 competent cells. The presence of the desired mutations was confirmed by DNA sequencing of the inserts. Site-directed variants were purified via chitin columns and DTT cleavage. Three mutants were obtained by cloning of their synthetic gene alleles into pTXB1. The activity of TagI variants was assayed on pBR322 (Dcm^+) plasmid as described above.

Error-prone PCR (Taq DNA polymerase in Mn^{2+} buffer, 35 cycles) was carried out to introduce random mutations into the *tagIR* gene. The mutated PCR DNA was inserted into pET28b (NdeI-XhoI, Kan^R) by NEB HI-FI assembly enzyme mix and subsequently transferred into NEB Turbo competent cells (Dcm^+) by transformation. The constitutive expression of WT TagI (6xHis tag at the C-terminus) in the Dcm^+ strain inflicts DNA damage and was toxic (lethal) to the host cells in transformation. Thus, only attenuated mutants survived the transformation. The 6xHis-tagged TagI variants were purified by nickel spin columns (Qiagen) from 20 ml of IPTG induced cells and their activity was assayed on pBR322 (Dcm^+).

Crystal structure determination

The TagI structure was solved by molecular replacement using the PHASER program (1). Due to the much higher level of confidence in the model, the SRA domain was placed before the HNH domain. The rotation function for the SRA domain was not very clear (log-likelihood gain of 23.7 and Z-score of 4.4 for the top solution, versus 22.4 and 4.1 for the next best solution), but the translation function was clear for space group P4(1)2(1)2 (log-likelihood gain 109.6, Z-score 13.1). For comparison, the Z-score in the alternative space groups was between 6.0 and 6.6, clearly indicating that the space group was P4(1)2(1)2. The HNH domain was harder to place. In P4(1)2(1)2, both at the rotation stage (log-likelihood gain 132.7, Z-score 4.4, compared to 131.8 and 3.9 for next solution) and the

translation stage (133.5 and 5.09, versus 133.1 and 5.06), the top solution stood out only weakly, but nevertheless proved to be correct.

The SRA domain could also be oriented and positioned using the automatic BALBES server (2) that also clearly selected the P4(1)2(1)2 space group (78% probability of a correct solution). The automatic model building based on the placed SRA domain of mouse UHRF1 (PDB code: 3f8j) resulted in the R and R_{free} values of approximately 40 and 48%. The SWISSMODEL built HNH domain model (and alternatively also the original ZRANB3 fragment template) could then be placed using the FFFEAR program (3). The solutions obtained by PHASER and BALBES/FFFEAR had consistent relative orientation of the two domains. We have next submitted the obtained composite model to iterative model building with the BUCCANEER (4) and ARP/wARP (5) programs which resulted in the 90% complete model and R and R_{free} of 21 and 27%. The missing part of the model included mainly the poorly ordered linker between the two domains. The model was completed and refined using the COOT (6) and REFMAC (7) programs. The refinement statistics and quality indicators are presented in Table S2. The final model parameters show that the resulting structure is very confident, despite the tentative initial steps of its determination. The atomic coordinates and the corresponding structure factors were deposited at the PDB with the 6GHS accession code.

Gel filtration and multi-angle light scattering

Size exclusion chromatography (SEC) was performed with the flow rate 0.5 ml/min in a buffer containing 15 mM Tris-HCl, pH 7.5, 200 mM NaCl, 0.5 mM EDTA, 1 mM DTT using the GE Healthcare Lifescience™ Superdex™ 200 Increase 10/300 GL Prepacked Tricorn™ Column connected to the NGC Scout 10 Medium-Pressure Liquid Chromatography System from Bio-Rad, RefractoMax 520 Refractive Index Detector from ERC Inc. and miniDAWN TREOS multi-angle light scattering system from Wyatt. The calibration curve was obtained using Gel Filtration Standard from Bio-Rad containing thyroglobulin (bovine) 670 kDa; gamma-globulin (bovine) 158 kDa; ovalbumin (chicken) 44 kDa and myoglobin (horse) 17 kDa. The concentration of TagI used in the assay was 75.5 μM (2.56 mg/ml). Light scattering data were analyzed using the Wyatt software based on the Zimm model (8,9). Mass dispersity was very low (1.001). The mass cited in the main text is the number averaged mass, but due to the low dispersity, the other masses are very similar.

Supplementary Figures

Fig. S1

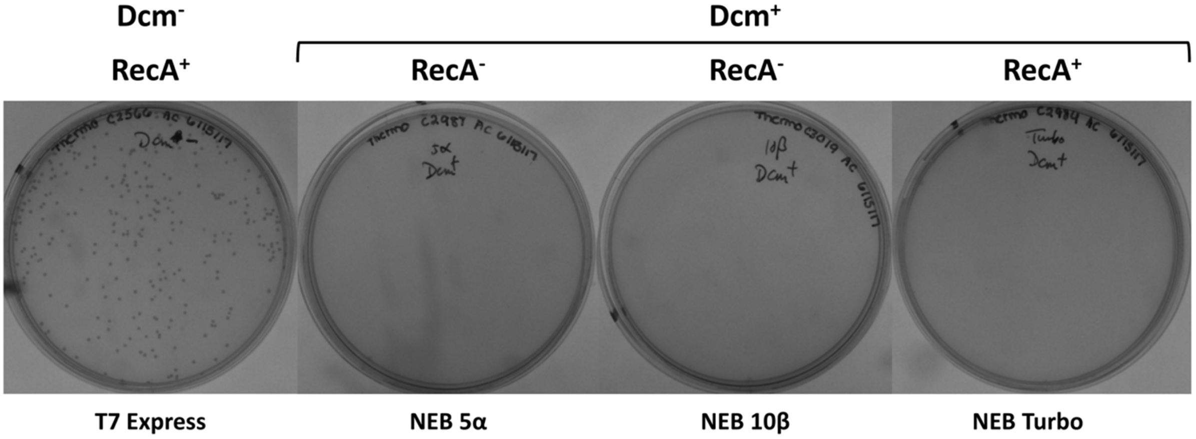

Fig. S1: TagI toxicity in Dcm$^+$ cells. pTXB1-*tagIR* (Amp^R) or pET28-*tagIR* (Kan^R) plasmids bearing *tagIR* gene were introduced into *E. coli* cells by DNA transformation. The plasmids proved to be toxic to Dcm$^+$ cells, and could only be transferred into the Dcm-deficient strains (e.g. T7 Express, C2566 or *E. coli* K strain C2925). No transformants were found in the recipient cells NEB 5α and 10β (RecA$^-$). The colonies on NEB Turbo recipient cells (RecA$^+$) were very small even after prolonged incubation.

Fig. S2

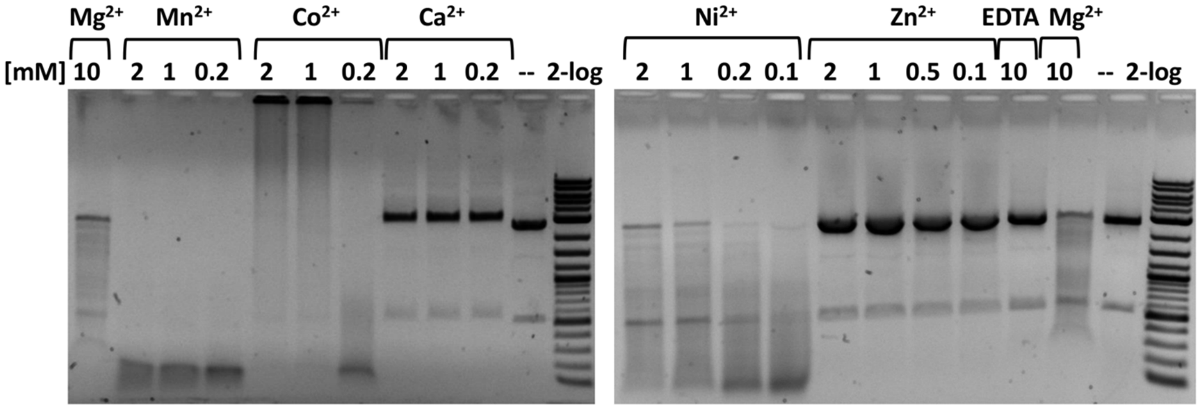

Fig. S2: Divalent metal ion requirement for TagI activity. 0.5 μg (~4.5 nM) PCR DNA fragments containing ^{5hm}C (3 and 0.5 kb) were digested by ~0.25 μg TagI (73.7 nM TagI dimer) in 50 μl of 1x buffer containing 0.1 M NaCl, 10 mM Tris-HCl, pH 7.5, 1 mM DTT, supplemented with divalent cations or EDTA, at 37 °C for 1 h. The reaction was stopped by the addition of 1.6 U of Protease K, followed by RE stop buffer after 15 min. TagI activity in divalent metal ions was estimated as: $Mn^{2+} > Co^{2+} > Ni^{2+} > Mg^{2+}$. TagI displays poor activity in Ca^{2+}, Zn^{2+} and EDTA.

Fig. S3

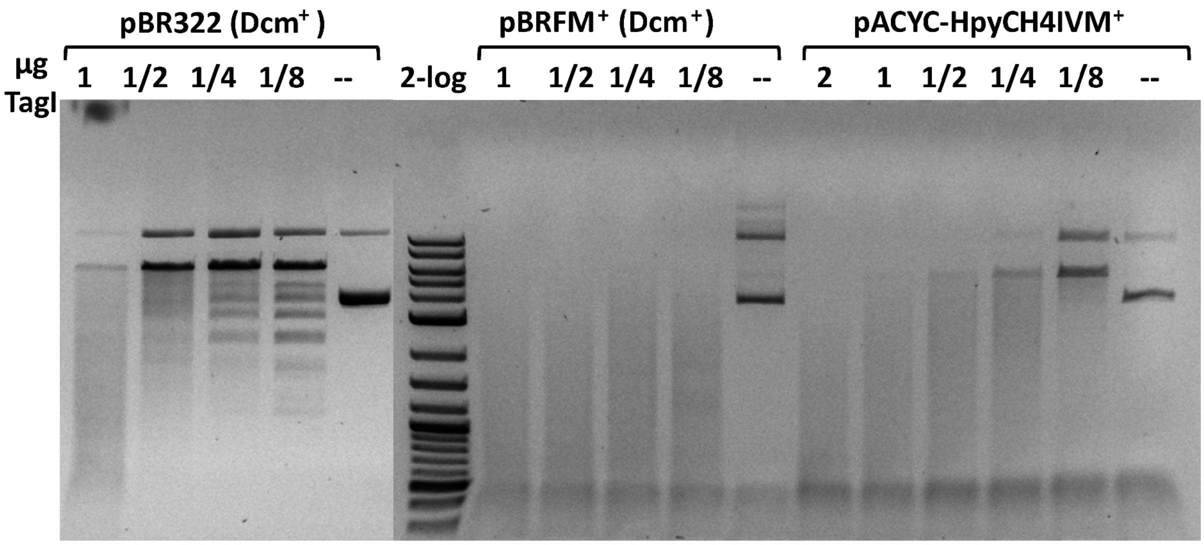

Fig. S3: Influence of the modified cytosine sequence context ($C^{5m}C$, $G^{5m}C$, $A^{5m}C$) on TagI activity. 0.5 µg of pBR322 (Dcm^+)(~3.5 nM), pBRFM$^+$ (Dcm^+)(~2.9 nM) and pACYC-HpyCH4IVM$^+$ (Dcm^-, ~3.0 nM) plasmid DNA was digested by TagI (in 2-fold dilution series) in NEB buffer 2.1 at 37 °C for 1 h. The amount of TagI protein in µg was shown on top of each lane, which corresponds 295, 147, 74, and 37 nM of TagI dimer, respectively. Inhibition of DNA cleavage by very high TagI concentration (compare lanes 1 and 2) is consistent with the requirement for more than one modified base for DNA cleavage.

Fig. S4

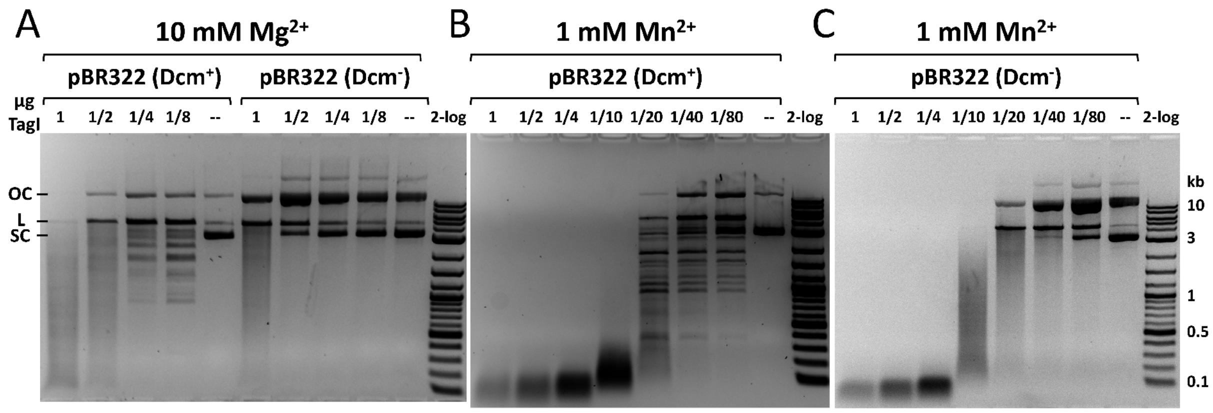

Fig. S4: TagI digestion of Dcm$^+$ and Dcm$^-$ pBR322 DNA in Mg^{2+} or Mn^{2+} buffer. 0.5 µg (~3.5 nM) of pBR322 plasmid was digested with decreasing concentrations of TagI. The buffer components were the same (NEB buffer 2.1) except for the divalent metal cations (10 mM $MgCl_2$ and 1 mM $MnCl_2$ final concentrations, respectively). The digestion was carried out at 37 °C for 1 h. 2-log DNA size marker (0.1 to 10 kb, NEB) was used. **(A)** In Mg^{2+} buffer TagI partially cleaved the modified pBR322 (Dcm$^+$, C^{5m}CWGG). At high enzyme concentration, it also digested unmodified DNA (Dcm$^-$) with accumulation of nicked and linear DNA, presumably due to the non-specific endonuclease activity. **(B,C)** In Mn^{2+} buffer the enzyme displayed higher specific activity and also cleaved unmodified DNA. At 50 ng (1/20 dilution, ~15 nM) to 12.5 ng enzyme (1/80 dilution, ~3.7 nM) digestions, specific cleavage products were clearly detected, suggesting the enzyme possesses certain sequence specificity. 1, 0.5, 0.25, 0.125 of TagI dimer in 50 µl reaction volume corresponds to 295, 147, 74, and 37 nM, respectively. SC: supercoiled; L: linear; OC: open circular plasmid DNA.

Fig. S5

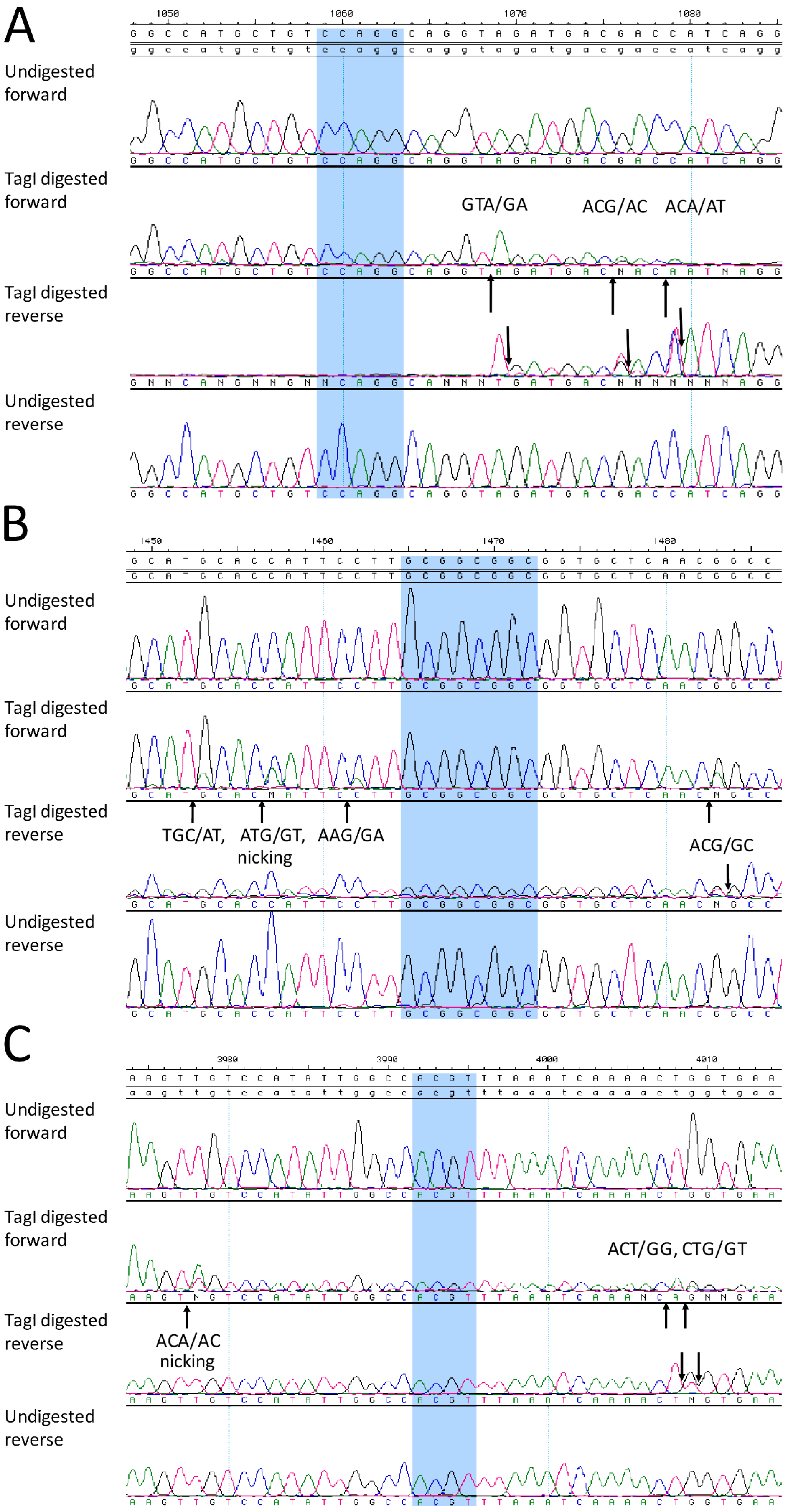
A
Undigested forward
TagI digested forward
GTA/GA
ACG/AC
ACA/AT
TagI digested reverse
Undigested reverse
B
Undigested forward
TagI digested forward
TagI digested reverse
TGC/AT,
ATG/GT,
nicking
AAG/GA
ACG/GC
Undigested reverse
C
Undigested forward
TagI digested forward
ACT/GG, CTG/GT
TagI digested reverse
ACA/AC
nicking
Undigested reverse

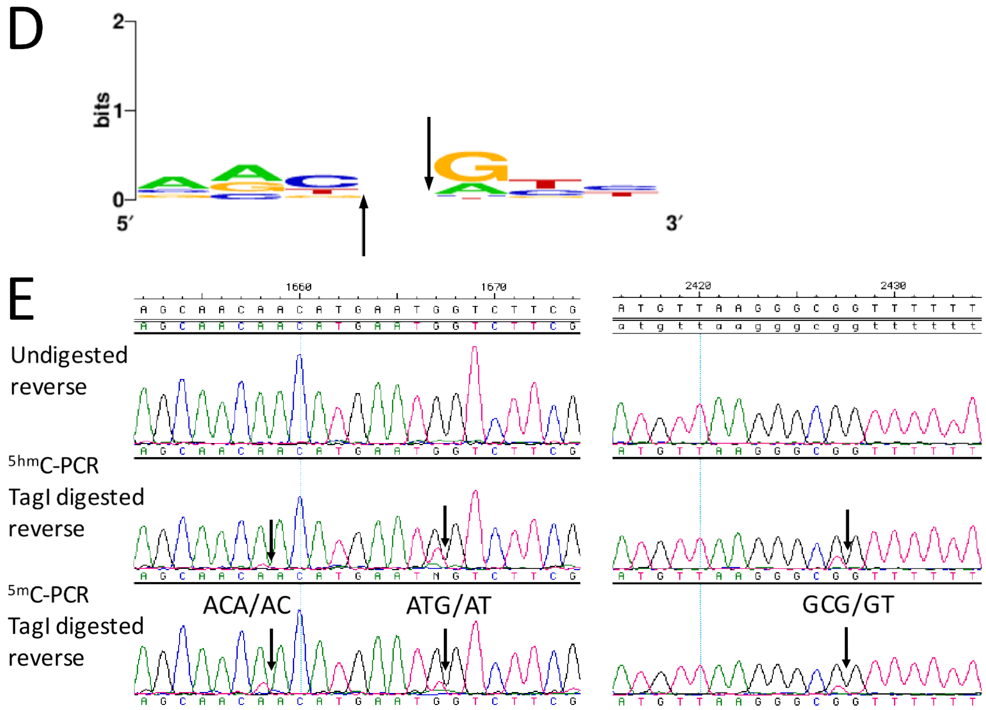

Fig. S5: Examples of sequence chromatograms from TagI digested DNA used to map the double-stranded cleavage/nicking sites. (A) Dcm-modified pBR322 plasmid ($C^{5m}CWGG$), **(B)** M.Fnu4HI modified pBRFM$^+$ plasmid ($G^{5m}CGGC$), **(C)** pACYC-HpyCH4IVM$^+$ plasmid ($A^{5m}CGT$) were digested by TagI at 50 °C. The modification sites are shaded blue. Arrows indicate cleavage (or nicking) site locations where doublets or extra high A or T peaks were detected (Taq DNA polymerase adds an extra A with template-independent terminal nucleotide transferase activity when it "runs-off" from a broken strand). The major cleavage/nicking sites were shown above the sequence chromatograms. **(D)** Cleavage site sequence logo from 20 double-stranded cuts in pBR322 (Dcm$^+$), pBRFM$^+$, and pACYC-HpyCH4IVM$^+$ (excluding nicked sites). The logo was derived from the following cut sites: GGTA/GAT, GACG/ACC, GACC/ATC, ACGG/GTG, CATG/ATC, CACG/GTC, AGGC/ATA, CATA/GGC, AACG/GCC, CCCA/GTC, AAGA/GGT, AGAG/GTT, AACC/TCT, ACCT/CTT, TGCT/GCA, AGCG/ACT, GACT/GCT, AGTT/GTC, AACT/GGT, ACTG/GTG. **(E)** TagI digestion of modified PCR DNA (amplified from pBR322) containing ^{5hm}C or ^{5m}C at 37 $^{\circ}$C.

Fig. S6

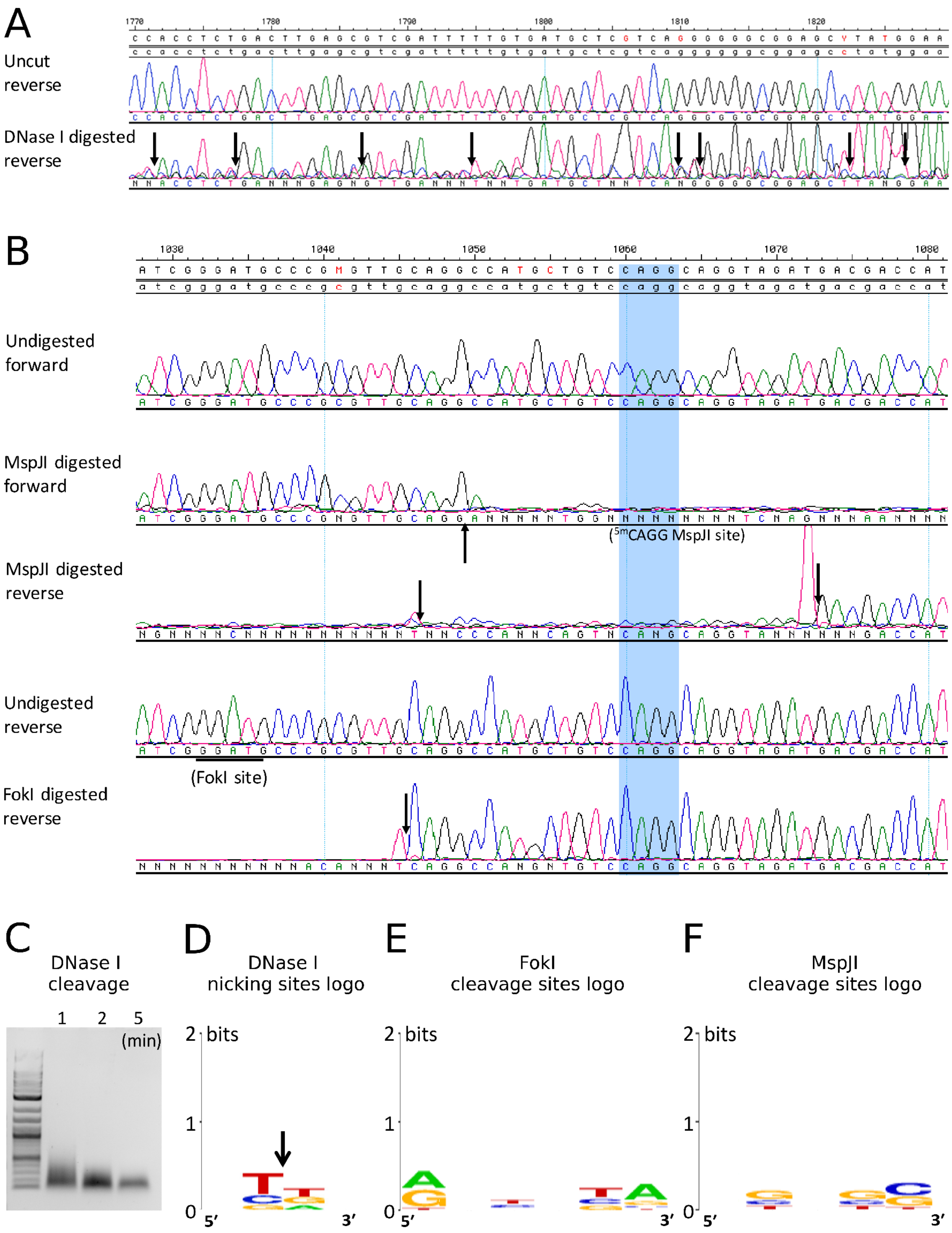

Fig. S6: Run-off sequencing of the control DNase I, FokI, and MspJI digests. (A) 5 µg pBR322 (17.5 nM) were digested by 0.1 U of diluted DNase I in the DNase I buffer at room temperature for 1, 2 and 5 min. **(B)** The FokI and MspJI digestions were carried out under the standard conditions. The cleavage products were subjected to run-off sequencing by Taq DNA polymerase. **(C)** The digestion products by DNase I. **(D-F)** The DNase I, FokI and MspJI cleavage site sequence logos were clearly different from the one observed for TagI, excluding the polymerase effects.

Fig. 7

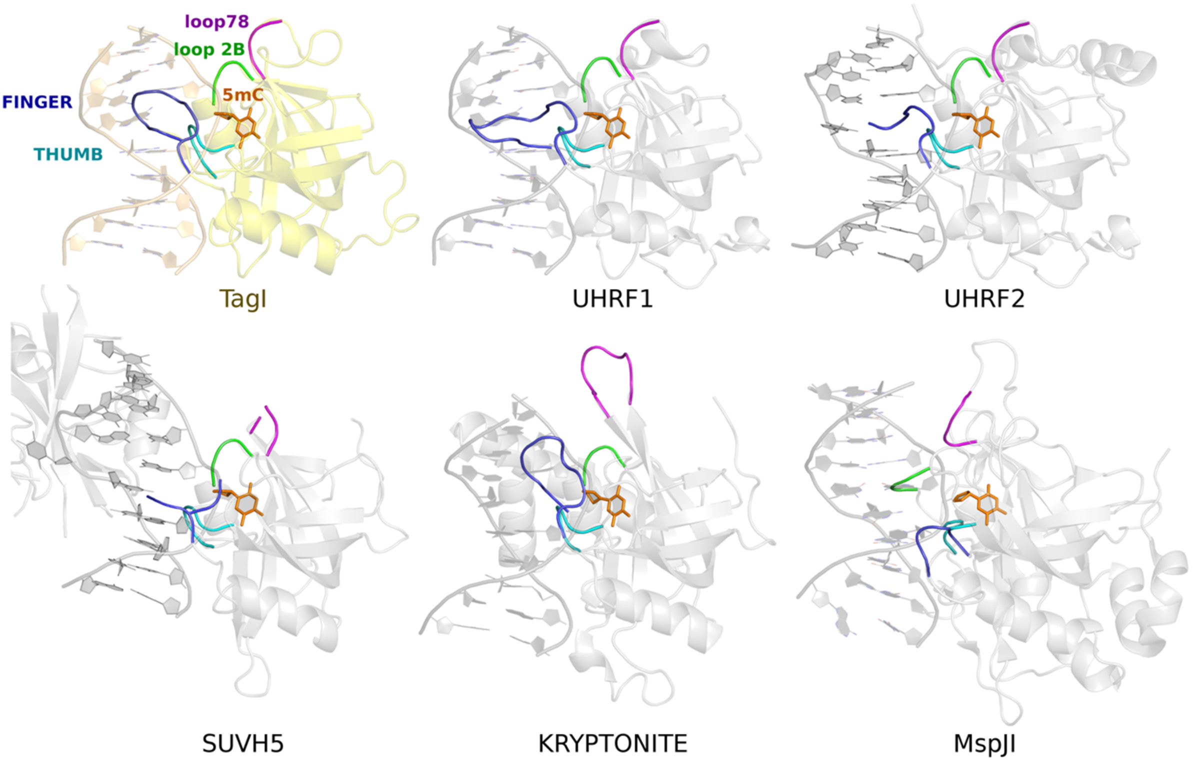

Fig. S7: Model of TagI SRA domain - DNA complex and structurally characterized SRA domain - DNA complexes of human UHRF1 and UHRF2 ubiquitin ligases (10,11), *A. thaliana* SUVH5 and KRYPTONITE histone methyltransferases (12,13), and *Mycobacterium* sp. MspJI restriction endonuclease (14). The binding mode of TagI to DNA was based on the human UHRF1 – DNA complex (PDB: 3clz (10)). The loops that were implicated in DNA binding are colored: finger – blue, thumb – cyan, loop 78 – magenta, loop 2B – green. The flipped base is indicated in orange.

Fig. S8

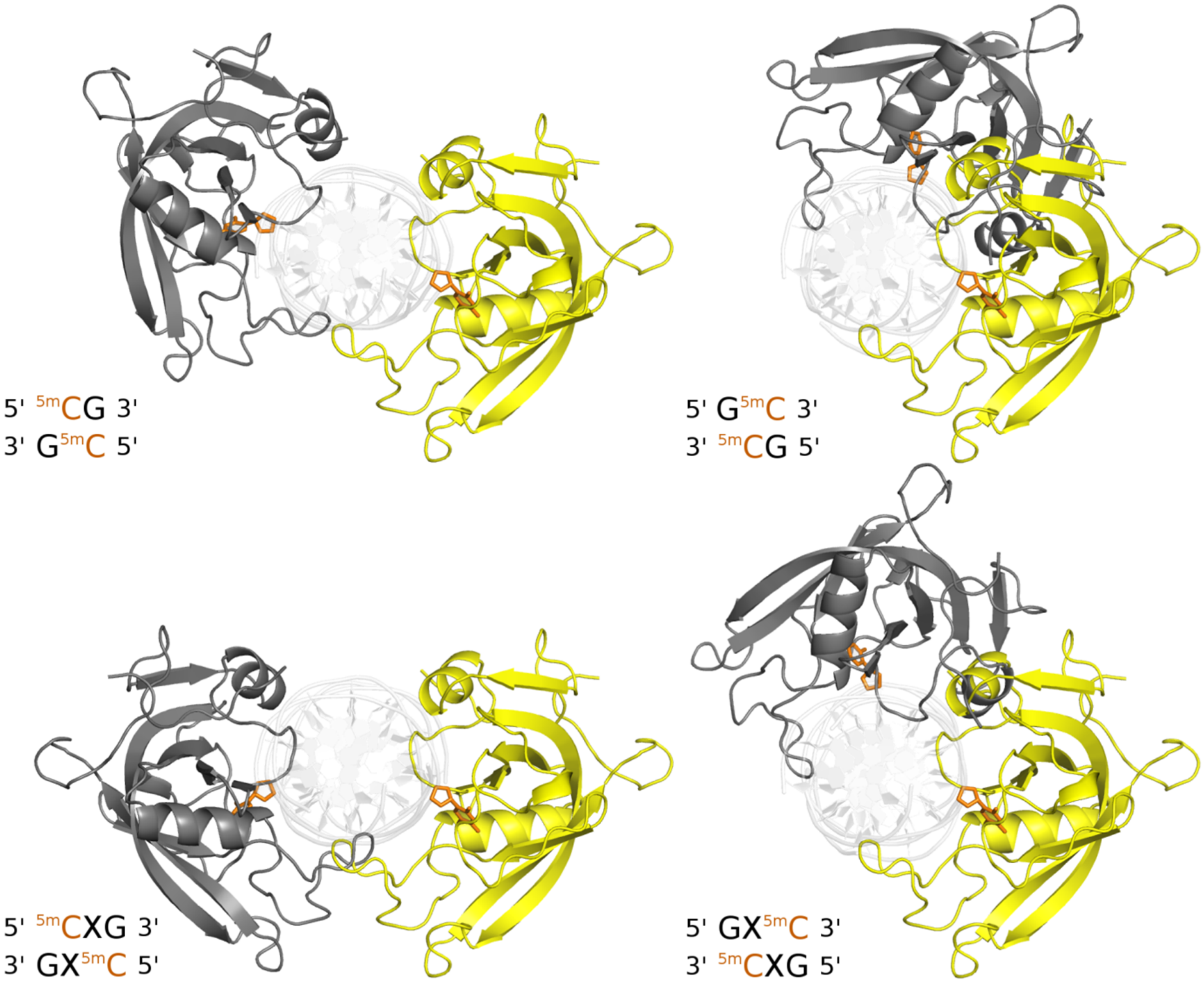

Fig. S8: Modelling of the binding of two SRA domains to a single DNA duplex. For DNA fully modified in some contexts such as G^{5m}C and GX^{5m}C, the two domains would clash and thus cannot simultaneously bind and flip the two modified cytosines. For other contexts such as ^{5m}CG and ^{5m}CXG, the steric conflicts are very mild and likely resolved by the minor adjustments of the loops. Thus in these contexts the two SRA domains of a TagI dimer can bind to fully modified DNA.

Fig. S9

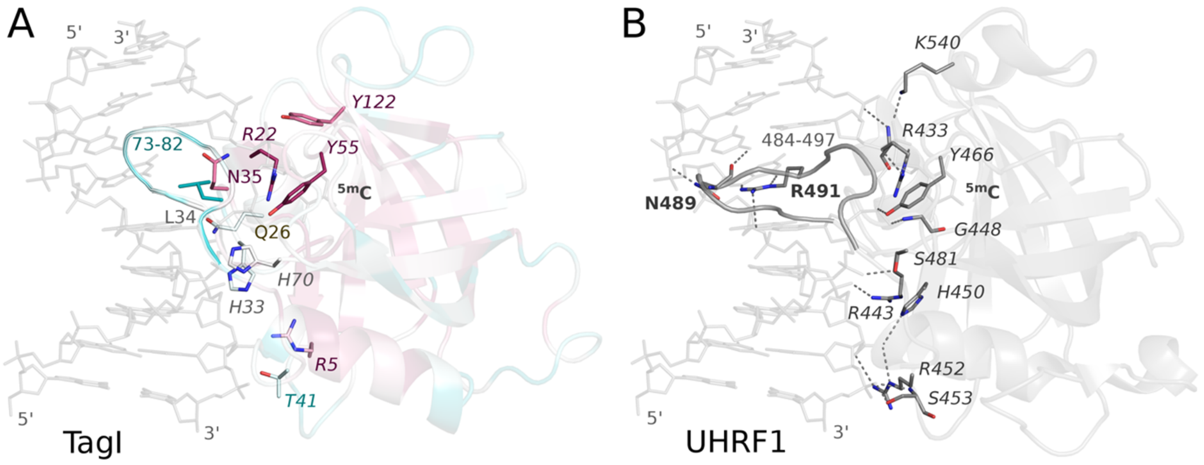

Fig. S9: Putative DNA binding residues of TagI (A) based on the experimentally determined ones of human UHRF1 (B) (2). TagI was colored according to the sequence conservation calculated by the ConSurf server (15). Amino acid residues predicted to be important for ^{5m}C recognition are highlighted with bold font for TagI SRA domain. The residues that are predicted for TagI and known for UHRF1 to interact with the DNA backbone are indicated with italic font. The DNA structure shown in (A) was not present in the crystal and is modelled based on the UHRF1 complex structure (10).

Fig. S10

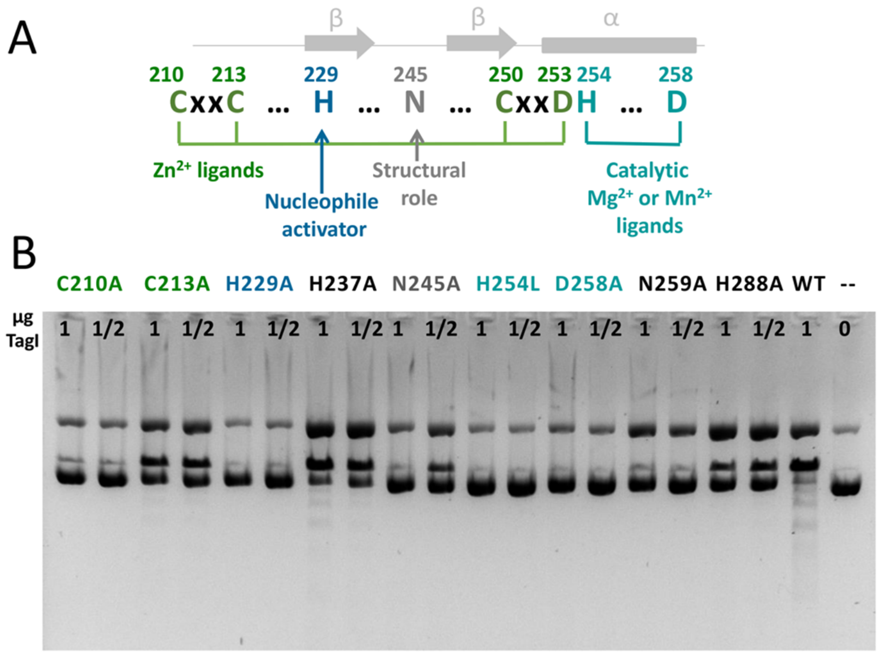

Fig S10: Schematic representation of the core region of the TagI HNH domain (A) and the effect of the substitution of functional residues on the enzyme activity (B). In the schematic representation, the moniker HNH residues are highlighted in bold and the roles of key residues are indicated. The secondary structure assignment shows the location of the active site residues with respect to the canonical ββα-Me motif of HNH endonucleases. H254L (6xHis-tagged, nickel column purified) was isolated by random mutagenesis, assayed by toxicity in Dcm^+ cells. Other mutant proteins 1 μg (~295 nM) and 0.5 μg (~147 nM) were purified from chitin columns and used to digest 1 μg (7 nM) of pBR322 (Dcm^+) at 37 °C for 1 h.

Fig. S11

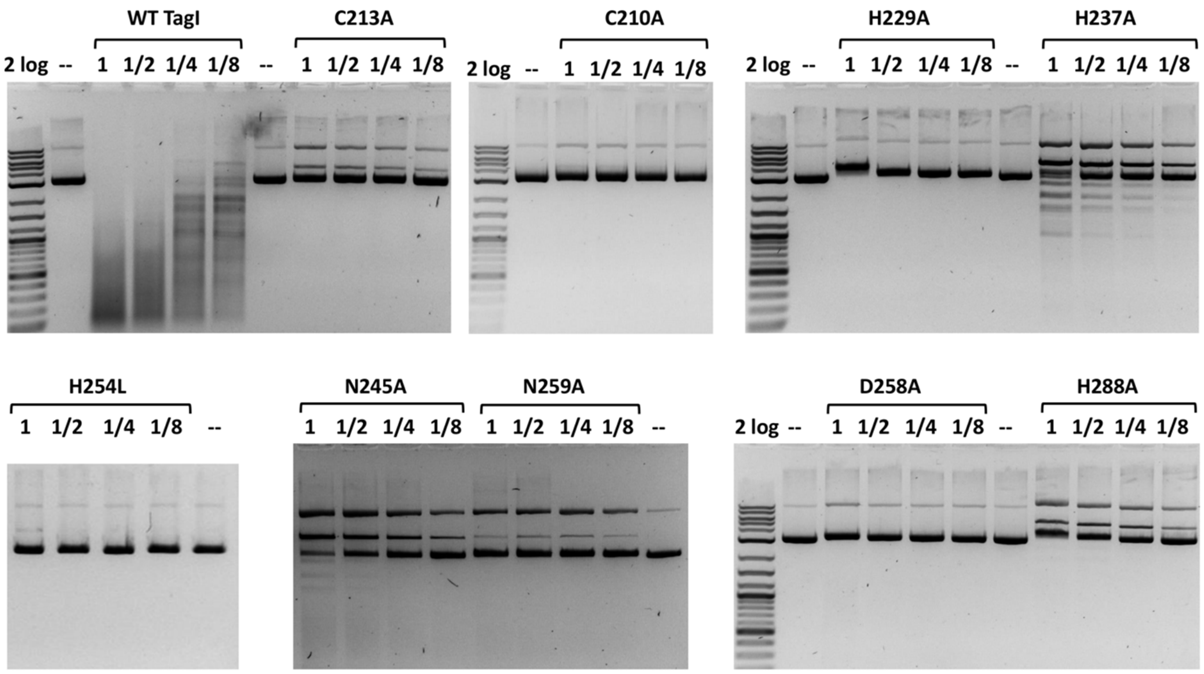

Fig. S11: Activity assay of TagI variants. Wild-type TagI and its variants were used to digest 1 μg (7.0 nM) of pBR322 (Dcm^+) DNA in NEB buffer 2.1 at 37 °C for 1 h. 1, 0.5, 0.25, and 0.125 μg of TagI dimer in 50 μl reaction volume correspond to 295, 147, 74, and 37 nM, respectively. Digested DNA was resolved in 0.8% agarose gels.

Fig. S12

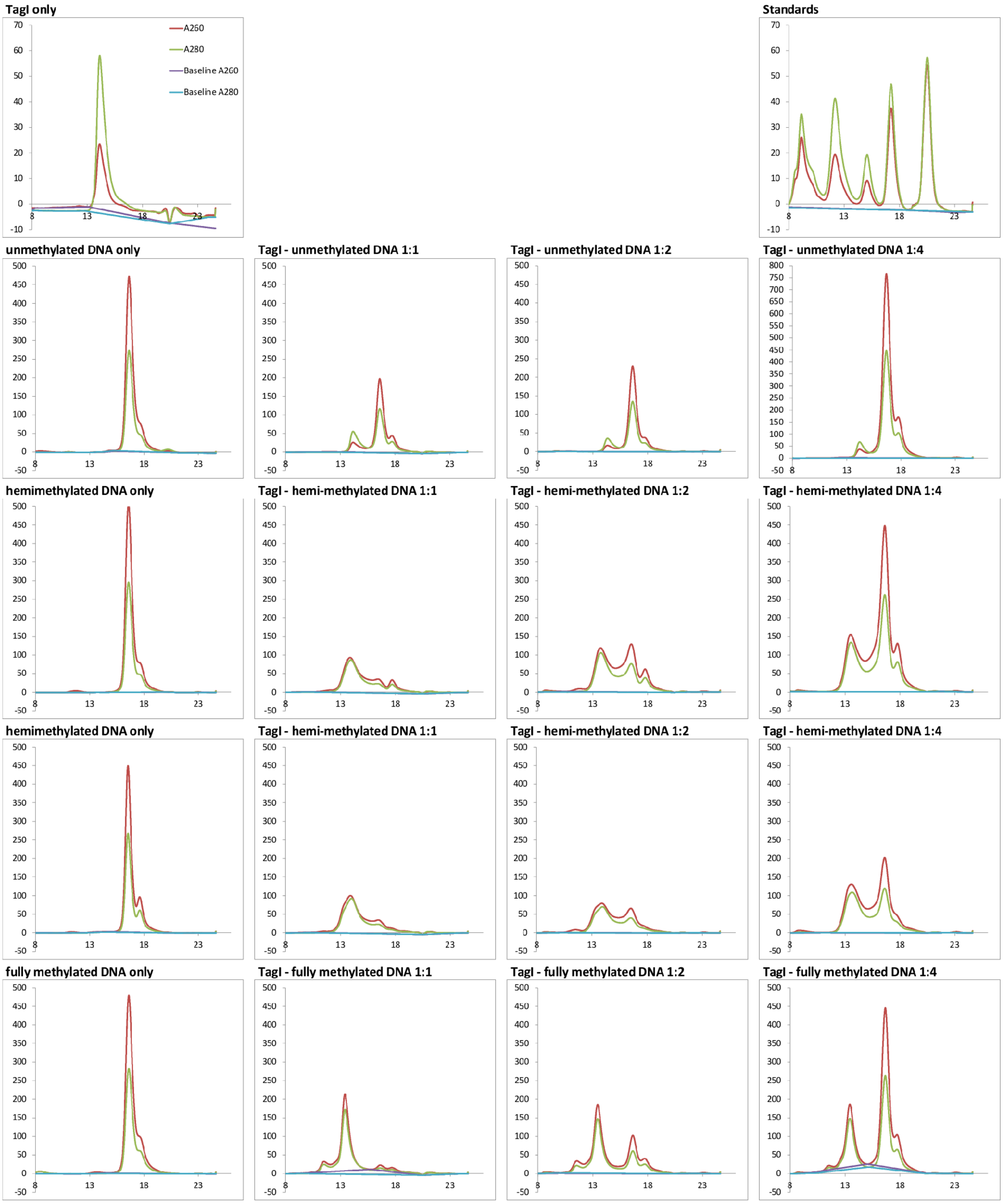

Fig. S12: Raw gel filtration data for TagI and dsDNA in various mixing ratios. TagI and unmodified, hemi- or fully methylated 17mer dsDNA were subjected to size exclusion chromatography either in separation or mixed together in various ratios. Two variants of hemimethylated oligoduplex were tested (with either top or bottom strand modified). The modification was introduced in the ^{5m}CWG context corresponding to Dcm modified DNA. TagI was mixed with DNA in either 1:1, 1:2 or 1:4 stoichiometry (protein dimer:dsDNA). Elution volumes are given in ml and absorbance is quoted in mAU (i.e. 0.001 $M^{-1}cm^{-1}$).

Fig. S13

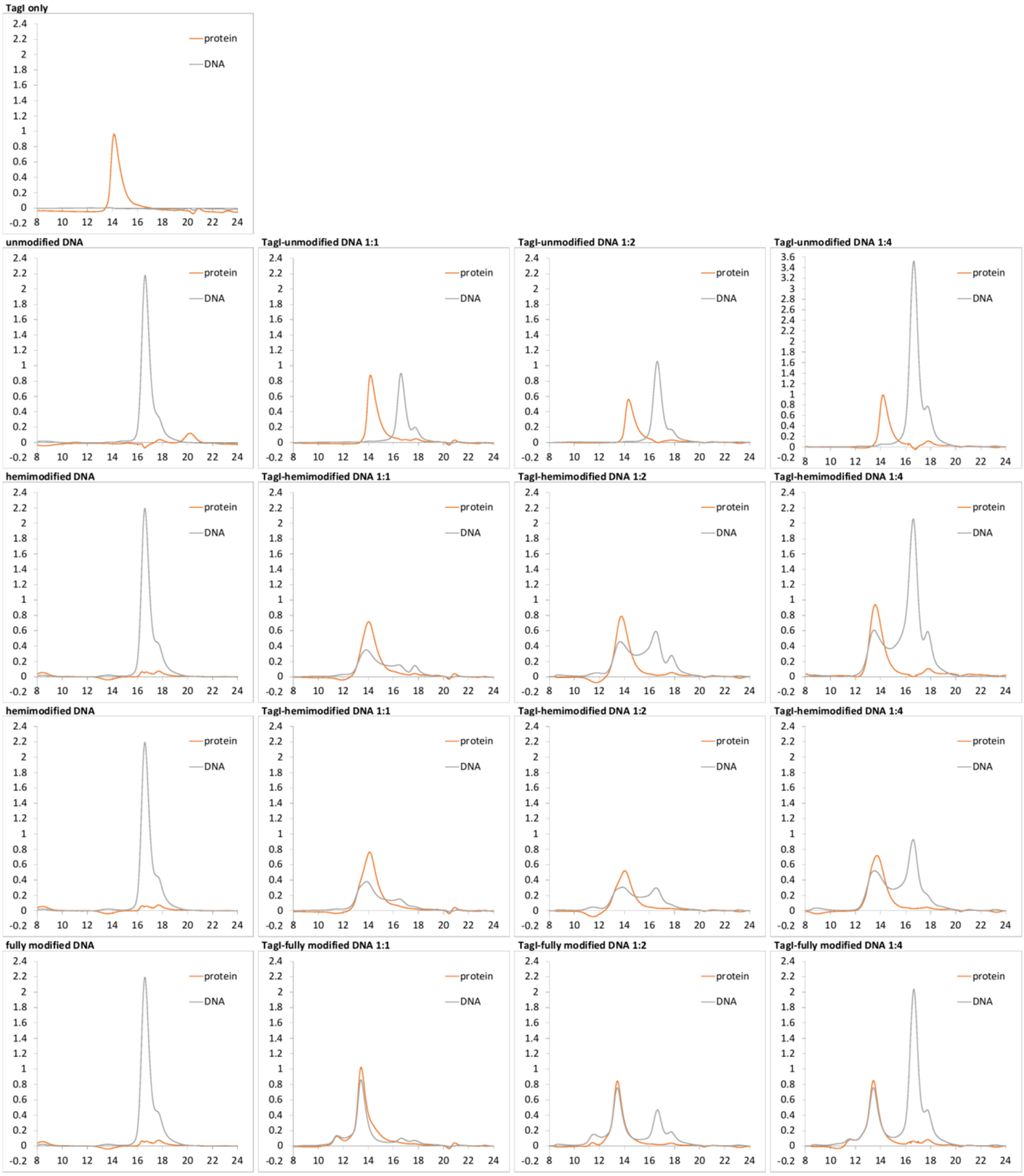

Fig. S13: Deconvoluted gel filtration data. UV absorbance data of Fig. S11 were used to concentrations of TagI dimer and of DNA duplexes. For the TagI dimer, we used a ProtParam (16) calculated 280 nm extinction coefficient of 60 740 $M^{-1}cm^{-1}$) and an OD_{280}/OD_{260} ratio of 2.42 derived from absorption data for TagI alone. For the DNA duplexes, we used an OligoCalc (17) calculated 260 nm extinction coefficient of 217 766 $M^{-1}cm^{-1}$, and an OD_{280}/OD_{260} ratio of 0.585 derived from absorption data for oligoduplexes alone. Elution volumes are given in ml and concentrations are given in µM.

Fig. S14

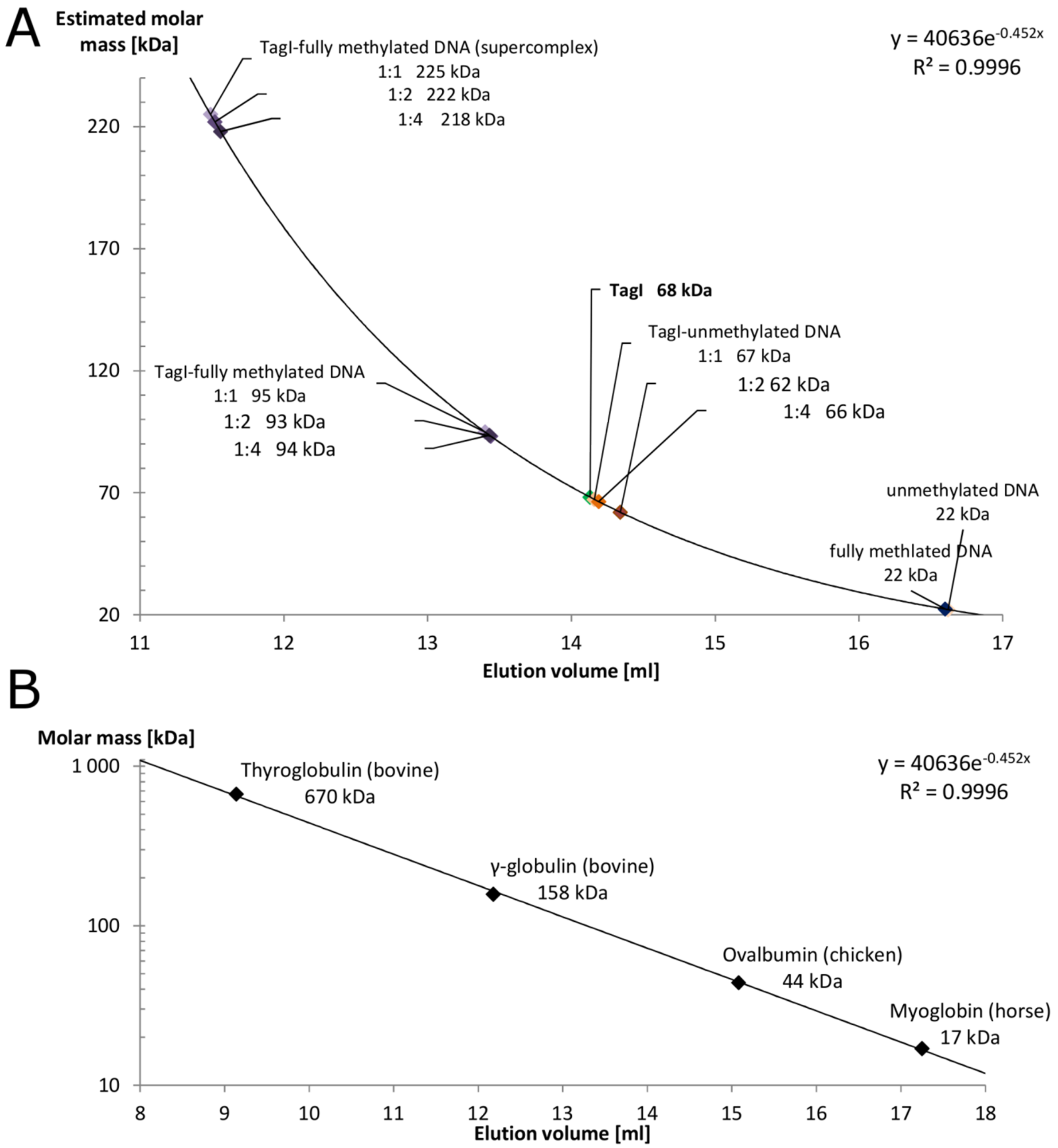

Fig. S14: Molecular mass of TagI and TagI-DNA complexes estimated from analytical gel filtration (A) and the calibration curve (B). For the interpolation, a linear dependence (with offset) between the elution volume and the logarithm of the molecular mass was assumed. Molecular masses for hemi-methylated complexes were not determined, because the elution peaks were broad, presumably due to a binding/unbinding equilibrium.

Fig. S15

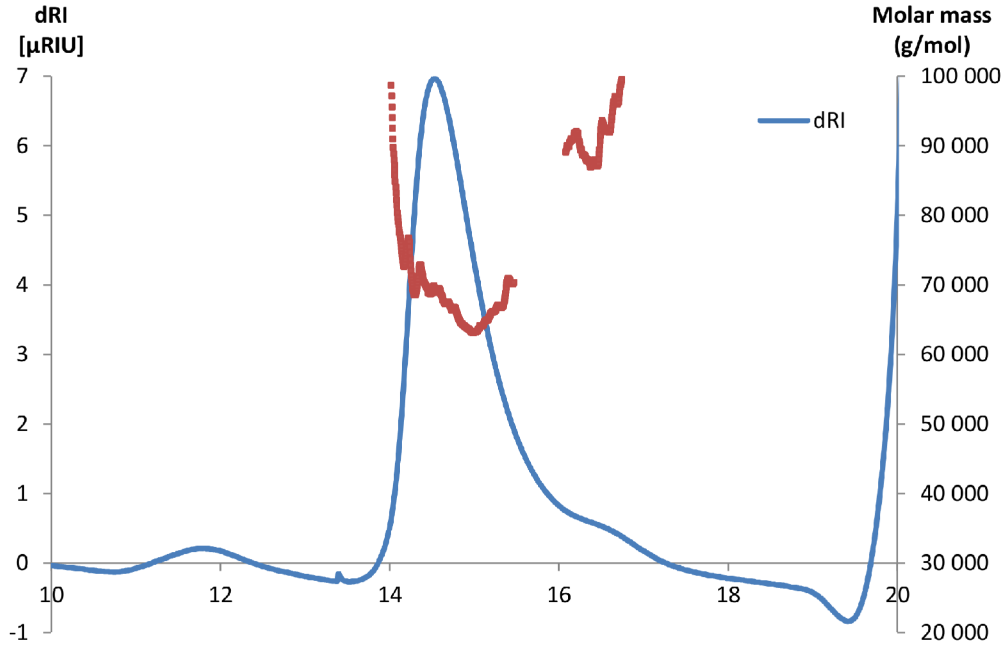

Fig. S15: Solution study of the TagI oligomeric state by SEC. Size exclusion chromatography combined with multi-angle light scattering was applied to determine the molecular mass of TagI in the absence of DNA. Differential refractive index was normalized for the protein peak. The resulting mass of 68 090 Da (±4%) agrees extremely well with the predicted mass of the protein dimer (67 872 Da).

Fig. S16

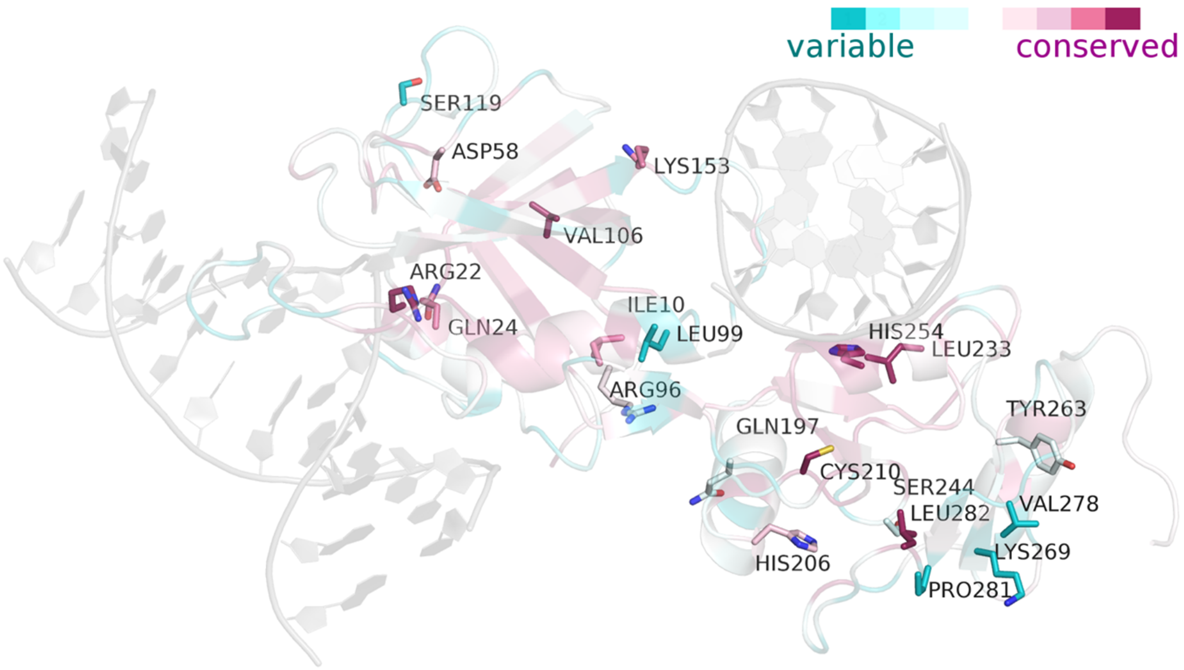

Fig. S16: Residues of TagI identified in an unbiased screen as important for activity. Alteration of the indicated residues reduced TagI toxicity to Dcm$^+$ *E. coli* cells. The complete list of obtained variants, phenotypes and structural context of the introduced mutations is presented in Table S3. Residues are presented in stick representation and colored according to the conservation scores determined using the ConSurf program (15). For the small number of inactivating mutations determined so far, a weak correlation with conservation scores is observed. Moreover, the mutations are rather evenly spread over the entire protein, and do not cluster in one region or on one face of it. For some mutations, the lack of activity is readily explained by the loss of key functional residues. Other mutations lie at the interface between the SRA and HNH domains in the crystal structure. As the significance of this interface in solution is uncertain, reduced or abolished activity may not be related to the location. Surprisingly many mutations map to protein surface. They could affect binding of DNA or of the linker between the SRA and HNH domains. Alternatively, they could be involved in inter-domain interactions of the domains for conformations not seen in the crystal.

Supplementary Tables

Table S1: Oligonucleotides used in this study.

**PCR primers to amplify *tagIR* gene for cloning into pET28b (NdeI-XhoI) and for error-prone PCR mutagenesis***	
Forward primer	5′ AGCGGCCTGGTGCCGCGCGGCAGCCATATGGCCTACAAACGTACTTTCGGG 3′
Reverse primer:	5′ TCAGTGGTGGTGGTGGTGGTGCTCGAGGGGAATGTTCGGAAGCTGGCTGCG 3′
**Sequencing primers for pBR322**	
F650	5′ CGTCGACCGATGCCCTTGAGAGC 3′
F1183	5′ GAACGGGTTGGCATGGATTGTAGG 3′
F2074	5′ GCGCGTTTCGGTGATGAC 3′
F2411	5′ ATCAGCTCACTCAAAGGCGGTAAT 3′
F4021	5′ TGTAACCCACTCGTGCACCCAACT 3′
R331	5′ TCCAAGTAGCGAAGCGAGCAG 3′
R660	5′ ATCGGTCGACGCTCTCCCTTATGC 3′
R1271	5′ CCGCCGGCTTCCATTCAGGT 3′
R1741	5′ GTCAATGCCAGCGCTTCGTTAATA 3′
R2896	5′ CATACCTCGCTCTGCTAATCCTGT 3′
R2751	5′ GGAGCGAACGACCTACACCGAACT 3′
**Sequencing primers for pBRFM$^+$ (pBR322 backbone with the *fnu4HIM* gene inserted into the BamHI and SphI sites)**	
F121	5′ CACCGTCACCCTGGATGCTGTA 3′
F1621	5′ TAAAGTCTGGAAACGCGGAAGTCA 3′
F1267(pBRFM$^+$)	5′ AAGCAGGAAATTCAGTTGTAGTG 3′
F2345(pBRFM$^+$)	5′ CATGATCGTGCTCCTGTCGTT 3′
F2658(pBRFM$^+$)	5′ ATCCATACCGCCAGTTGTTTACCC 3′
F3113(pBRFM$^+$)	5′ ACGTAGCGATAGCGGAGTGTA 3′
F4017(pBRFM$^+$)	5′ CTTTGATCTTTTCTACGGGGTCTG 3′
R642(pBRFM$^+$)	5′ CTTTTCTATAACCTGCTACACTAA 3′
R840	5′ AGCGACAGGCCGATCGTCGCG 3′
R2600(pBRFM$^+$)	5′ TGCCAGCGCTTCGTTAATACAGAT 3′
R2784(pBRFM$^+$)	5′ GGGGATTTCTGTTCATGGGGGTAA 3′
R3916(pBRFM$^+$)	5′ TGCCGGATCAAGAGCTACCAACT 3′
R5150(pBRFM$^+$)	5′ ATGTGCGCGGAACCCCTATTTGT 3′

**Sequencing primers pACYC-HpyCH4IVM**[+]	
F45	5′ AGGCCGGATAAAACTTGTGCT 3′
F2783	5′ CGGCACCTCGCTAACGGATTCACC 3′
F3679	5′ CGACCCTGCCCTGAACCGACGAC 3′
R181	5′ ATCCCAATGGCATCGTAAAGAACA 3′
R594	5′ CACTCCGCTAGCGCTGATGTCC 3′
R3472	5′ GGGTAGCCAGCAGCATCCT 3′
**Oligos for TagI binding and gel filtration assay**	
top strand unmodified	5′ CCTAGAGCAGCAATCCC 3′
top strand ^{5m}C-modified	5′ CCTAGAG^{5m}CAGCAATCCC 3′
bottom strand unmodified	5′ GGGATTGCTGCTCTAGG 3′
bottom strand ^{5m}C-modified	5′ GGGATTG^{5m}CTGCTCTAGG 3′
**Oligos for TagI activity assay**	
top strand unmodified	5′ ATGCAGAACAAG**C**CGAATTAATAGGCGGCCGAAGC TTATAG**C**ATTGAT 3′
top strand with both sites ^{5m}C-modified	5′ ATGCAGAACAAG^{5m}CCGAATTAATAGGCGGCCGAAG CTTATAG^{5m}CATTGAT 3′
bottom strand unmodified	5′ ATCAATGCTATAAGCTTCGGCCGCCTATTAATTCGG CTTGTTCTGCAT 3′
bottom strand with both sites ^{5m}C-modified	5′ ATCAATG^{5m}CTATAAGCTTCGGCCGCCTATTAATTCG G^{5m}CTTGTTCTGCAT 3′
**Primers for site-directed mutagenesis of Zn^{2+} binding site and putative catalytic residues**[$]	
C210A forward	5′ GCTCAGGTATGTGGCATTGTAATT 3′
C210A reverse	5′ TCTATGTTTGTGCCACCCTTTTAC 3′
C213A forward	5′ GCTGGCATTGTAATTGAAGTGGAT3′
C213A reverse	5′ TACCTGACATCTATGTTTGTGCCA 3′
H229A forward	5′ GCTATACGGCCCCTTGGCAGAAAG 3′
H229A reverse	5′ AGCACCCTGCGAATAAGGACCGAC 3′
H237A forward	5′ GCCGGTGGGCCAGACGTGGAGTCC 3′
H237A reverse	5′ CTTTCTGCCAAGGGGCCGTATATG 3′
H288A forward	5′ GCCGTAATCGATTTGGATCATATA 3′
H288A reverse	5′ ACGGGGATGCACTCTAAGGGGGCC 3′

[*] Vector sequence underlined.
[$] TagI mutants N245A, D258A and N259A were constructed using gene blocks containing the desired mutation.

Table S2: Data collection and refinement statistics.

Data collection statistics	
Space group	P4(1)2(1)2
Cell dimensions<br>a (Å)<br>c (Å)	 72.96<br>207.58
Wavelength (Å)	1. 54178
Resolution range (Å)<br>Highest shell<br>Lowest shell	20 - 2.92<br>20 - 11.4<br>3.02 - 2.92
Total reflections	42004
Unique reflections	12496
Completeness (%)*	96.1 (63.9, 96.2)
Multiplicity *	3.2 (1.7, 3.3)
Mean $I/\sigma I$ *	4.4 (8.8, 1.20)
R(sig) (%)*	21.96 (11.1, 94.85)
R(merge) (%)*	23.86 (6.7, 69.8)
Solvent content (%)	70
B(iso) from Wilson (Å^2)	53.5
Refinement statistics	
Protein atoms excluding H	2278
Solvent molecules	53
R_{cryst} (%)	19.24
R_{free} (%)#	22.31
RMSD bond lengths (Å)	0.008
RMSD angles (°)	1.34
Ramachandran favored region (%)	98.2
Ramachandran allowed region (%)	100.0
Molprobity clash score	0.5

* Lowest and highest shell in brackets
# 5% of reflections were set aside randomly

Table S3: TagI variants selected via random mutagenesis by error-prone PCR and selection against endonuclease activity. The conservation score is calculated by ConSurf server (15). The scores are normalized to average score for all amino acids in the protein as zero, and the standard deviation one. The more conserved a position, the lower (more negative the score). The location of mutation sites in the structure is presented in Fig. S16.

Mutation	Nicking activity	Conservation score	Residues observed in the alignment	Predicted role	Structural position
Q24R	+/-	-0.86	KHDQGEA	unclear	solvent exposed
**L282H**	**+**	**-1.23**	**L**	**structural, HNH**	**hydrophobic cluster**
V106A	+/-	-1.27	LVMI	unclear	buried
K153I	+/-	-1.05	RQKTS	unclear	solvent exposed
**D58G**	**+/-**	**-0.66**	**HVKNDFSAE**	**functional, SRA**	**binds the Watson-Crick edge of the flipped base**
K99S	+/-	1.40	KLIDFQCEVHYNTSRA	unknown	domain interface in the crystal
Q197R	+/-	0.31	SRAEKVQN	unclear	solvent exposed
**C210S**	**+**	**-1.15**	**C**	**functional, HNH**	**Zn^{2+} ligand**
I10T	+	-0.82	GILVACPT	unclear	partially exposed
R96G	+	-0.58	EARTGNIYDVK		domain interface in the crystal
T188S	++	0.16	TRDLFQWKSMVNIEAH	unclear	interdomain linker
**H206R**	**++**	**-0.52**	**HY**	**structural, HNH**	**hydrogen bonding cluster**
K269R	+	2.62	GDQLKVEARST	unclear	solvent exposed
S119P	+	2.27	LKVNGIQDSPTEA	unclear	solvent exposed
**L233P**	**+**	**-1.23**	**L**	**functional, HNH**	**packs against His229 HNH nucleophile activator**
**H254L**	**+**	**-1.15**	**HR**	**functional, HNH**	**active site metal ligand**
STOP	+				
**R22H**	**+**	**-1.14**	**RQFWKM**	**functional, SRA**	**phosphate binding**
Y263N	+	0.20	VFYIGTSA	unclear	solvent exposed
S244P	+++	0.67	AEGNDST	unclear	solvent exposed
V278G	+++	1.93	IQVMLRETS	unclear	solvent exposed
P281H	+++	2.49	PSERHVKDQ	unclear	solvent exposed

References

1. Mccoy, A.J., Grosse-Kunstleve, R.W., Adams, P.D., Winn, M.D., Storoni, L.C. and Read, R.J. (2007) Phaser crystallographic software. *Journal of Applied Crystallography*, **40**, 658-674.
2. Long, F., Vagin, A.A., Young, P. and Murshudov, G.N. (2008) BALBES: a molecular-replacement pipeline. *Acta Crystallogr D Biol Crystallogr*, **64**, 125-132.
3. Cowtan, K. (1998) Modified phased translation functions and their application to molecular-fragment location. *Acta Crystallogr D Biol Crystallogr*, **54**, 750-756.
4. Cowtan, K. (2006) The Buccaneer software for automated model building. 1. Tracing protein chains. *Acta Crystallographica Section D-Biological Crystallography*, **62**, 1002-1011.
5. Perrakis, A., Harkiolaki, M., Wilson, K.S. and Lamzin, V.S. (2001) ARP/wARP and molecular replacement. *Acta Crystallographica Section D-Biological Crystallography*, **57**, 1445-1450.
6. Emsley, P. and Cowtan, K. (2004) Coot: model-building tools for molecular graphics. *Acta Crystallographica Section D-Biological Crystallography*, **60**, 2126-2132.
7. Murshudov, G.N., Skubak, P., Lebedev, A.A., Pannu, N.S., Steiner, R.A., Nicholls, R.A., Winn, M.D., Long, F. and Vagin, A.A. (2011) REFMAC5 for the refinement of macromolecular crystal structures. *Acta Crystallographica Section D-Biological Crystallography*, **67**, 355-367.
8. Zimm, B.H. (1948) The Scattering of Light and the Radial Distribution Function of High Polymer Solutions. *J Chem Phys*, **16**, 1093-1099.
9. Wyatt, P.J. (1993) Light-Scattering and the Absolute Characterization of Macromolecules. *Anal Chim Acta*, **272**, 1-40.
10. Avvakumov, G.V., Walker, J.R., Xue, S., Li, Y., Duan, S., Bronner, C., Arrowsmith, C.H. and Dhe-Paganon, S. (2008) Structural basis for recognition of hemi-methylated DNA by the SRA domain of human UHRF1. *Nature*, **455**, 822-825.
11. Zhou, T., Xiong, J., Wang, M., Yang, N., Wong, J., Zhu, B. and Xu, R.M. (2014) Structural basis for hydroxymethylcytosine recognition by the SRA domain of UHRF2. *Mol Cell*, **54**, 879-886.
12. Rajakumara, E., Law, J.A., Simanshu, D.K., Voigt, P., Johnson, L.M., Reinberg, D., Patel, D.J. and Jacobsen, S.E. (2011) A dual flip-out mechanism for 5mC recognition by the Arabidopsis SUVH5 SRA domain and its impact on DNA methylation and H3K9 dimethylation in vivo. *Genes Dev*, **25**, 137-152.
13. Du, J., Johnson, L.M., Groth, M., Feng, S., Hale, C.J., Li, S., Vashisht, A.A., Wohlschlegel, J.A., Patel, D.J. and Jacobsen, S.E. (2014) Mechanism of DNA methylation-directed histone methylation by KRYPTONITE. *Mol Cell*, **55**, 495-504.
14. Horton, J.R., Wang, H., Mabuchi, M.Y., Zhang, X., Roberts, R.J., Zheng, Y., Wilson, G.G. and Cheng, X. (2014) Modification-dependent restriction endonuclease, MspJI, flips 5-methylcytosine out of the DNA helix. *Nucleic Acids Res*, **42**, 12092-12101.
15. Ashkenazy, H., Abadi, S., Martz, E., Chay, O., Mayrose, I., Pupko, T. and Ben-Tal, N. (2016) ConSurf 2016: an improved methodology to estimate and visualize evolutionary conservation in macromolecules. *Nucleic Acids Res*, **44**, W344-350.
16. Artimo, P., Jonnalagedda, M., Arnold, K., Baratin, D., Csardi, G., de Castro, E., Duvaud, S., Flegel, V., Fortier, A., Gasteiger, E. *et al.* (2012) ExPASy: SIB bioinformatics resource portal. *Nucleic Acids Res*, **40**, W597-603.
17. Kibbe, W.A. (2007) OligoCalc: an online oligonucleotide properties calculator. *Nucleic Acids Res*, **35**, W43-46.
